# Atrial dysfunction: a contrast-free marker for HFpEF in obese diabetics—insights from comprehensive CMR and serum biomarker analyses

**DOI:** 10.1186/s12933-025-02808-3

**Published:** 2025-06-18

**Authors:** Rebecca Elisabeth Beyer, Maximilian Leo Müller, Patrick Doeblin, Stefanie Maria Werhahn, Amedeo Chiribiri, Carsten Tschöpe, Smita Sampath, G. Brandon Atkins, Dawn Cislak, An Bautmans, John Palcza, Tom McAvoy, Asad Abu Bakar, Anita Y. H. Lee, Xuemei Zhao, Maximilian G. Posch, Johannes Wieditz, Radu Tanacli, Victoria Zieschang, Mithal Nassar, Seyedeh Mahsa Zamani, Christian Stehning, Frank Edelmann, Djawid Hashemi, Sebastian Kelle

**Affiliations:** 1 Department of Cardiology, Angiology and Intensive Care Medicine, Deutsches Herzzentrum der Charité– Medical Heart Center of Charité and German Heart Institute Berlin, Campus Virchow-Klinikum, Augustenburger Platz 1, 13353 Berlin, Germany; 2 Charité– Universitätsmedizin Berlin, corporate member of Freie Universität Berlin and Humboldt Universität Zu Berlin, Charitéplatz 1, 10117 Berlin, Germany; 3 DZHK (German Centre for Cardiovascular Research), Partner Site Berlin, Berlin, Germany; 4 Berlin Institute of Health (BIH) at Charite and Berlin-Berlin Brandenburger Center for Regenerative Therapies (BCRT), Berlin, Germany; 5 Philips Clinical Science, Hamburg, Germany; 6 Quantitative Biosciences, MSD, Singapore, Singapore; 7 Merck & Co., Inc., Rahway, NJ USA; 8 School of Biomedical Engineering and Imaging Sciences, King’s College London, BHF Centre of Excellence and the NIHR Biomedical Research Centre at Guy’s and St Thomas’ NHS Foundation Trust, The Rayne Institute, St Thomas’ Hospital, London, UK; 9 Charité Research Organisation GmbH, Berlin, Charitéplatz 1, 10117 Berlin, Germany; 10 Department of Medical Statistics, University Medical Center Göttingen, Göttingen, Germany

**Keywords:** HFpEF, Obesity, Type 2 diabetes mellitus, Cardiac magnetic resonance, Left atrial strain, IL1RL1, Biomarkers

## Abstract

**Background:**

The diagnostic criteria for HFpEF remain inconsistently defined, further confounded by comorbidities such as obesity and type 2 diabetes mellitus (T2DM), which are thought to contribute to its pathogenesis via chronic pro-inflammatory mechanisms. This study aimed to evaluate the relationship between advanced cardiac magnetic resonance (CMR) imaging and pro-fibrotic and inflammatory serum biomarkers, assessing their potential to discriminate HFpEF from associated comorbid conditions.

**Methods:**

This was an exploratory analysis of a prospective cohort study of 35 obese/overweight participants (mean age 64 ± 8 years, 23% females), including 16 with T2DM, 13 with HFpEF (NYHA II–III) and T2DM, and 6 healthy controls. All subjects underwent comprehensive contrast-enhanced CMR at a 3 T scanner (Philips Ingenia, The Netherlands), including assessment of left ventricular and left atrial (LA) volumetry and function, myocardial perfusion reserve (MPR), and diffuse fibrosis imaging (ECV). Obtained serum biomarkers were Pentraxin-3, Galectin-3 and Interleukin-1 Receptor-Like 1 (IL1RL1). Statistical analyses included one-way ANOVA, Tukey test, Pearson’s correlation, regression and receiver operating characteristic analyses, and intra-class correlation.

**Results:**

In multivariable regression, impaired measures of LA structure and function emerged as the only independent discriminators of HFpEF, with LA maximum volume showing an OR of 1.13 (95% CI 1.05–1.28), reservoir strain of 0.71 (95% CI 0.44–0.89), conduit strain of 0.57 (95% CI 0.32–0.82) and booster strain of 0.70 (95% CI 0.48–0.89) per unit increase. No differences in MPR nor ECV were observed between the groups. While serum biomarkers Galectin-3 and Pentraxin-3 were significantly higher in HFpEF vs. obese controls (16.1 ng/ml ± 3.8 ng/ml vs. 10.6 ng/ml ± 3.7 ng/ml, *p* = 0.011, and 0.84 ng/ml ± 0.67 ng/ml vs. 0.21 ng/ml ± 0.05 ng/ml, *p* = 0.031, respectively), these biomarkers remained within normal limits and showed only moderate correlations with CMR metrics. Highest inter-study reproducibility was seen in MPR (ICC: 0.94), LA Reservoir Strain (ICC: 0.84) and serum biomarkers (ICC: 0.087–0.93).

**Conclusion:**

CMR markers of diffuse fibrosis and microvascular dysfunction may not differentiate HFpEF from obese or diabetic controls. However, left atrial function assessment may evolve to be a reproducible and practical CMR marker, effectively distinguishing HFpEF independent of fibrotic remodeling.

**Supplementary Information:**

The online version contains supplementary material available at 10.1186/s12933-025-02808-3.

## Introduction

Heart failure with preserved ejection fraction (HFpEF) is a growing global health concern, affecting an estimated 1–3% of the adult population [1]. Current guidelines base diagnosis on the specific criteria, including the nature of symptoms and signs observed during physical examination, objective evidence of cardiac structural and/or functional abnormalities consistent with impaired left ventricular (LV) diastolic function or elevated filling pressures, and a left ventricular ejection fraction (LVEF) exceeding 50% [2]. However, these diagnostic criteria are not uniformly defined, and even scoring systems endorsed by the major cardiac societies still carry substantial diagnostic uncertainty [2–5]. This ambiguity likely reflects an incomplete understanding of the HFpEF pathophysiology, as well as regional differences observed in patient characteristics and phenotypes [6].

The diagnostic challenge is further compounded by the often non-specific and varying nature of symptoms, many of which overlap significantly with those of associated comorbidities. Many of these comorbidities are believed to contribute gradually and meaningfully to the development and progression of HFpEF through inflammatory and pro-fibrotic pathways [6, 7]. Paulus et al. introduced an emerging paradigm of HFpEF in which systemic inflammation leads to activation of the microvascular endothelium, resulting in increased myocardial stiffness, interstitial fibrosis, and ultimately myocardial dysfunction. As a result, biomarkers of fibrosis and inflammation have received increased attention due to their potential mechanistic involvement and their association with disease characteristics that are commonly, but not exclusively, observed in patients with HFpEF [2, 7–9]. Among others, Pentraxin-3 (PTX3) has sparked interest for its linkage to vascular inflammation and impaired endothelial nitric oxide synthesis, mechanisms which have been shown to drive microvascular dysfunction and subsequent cardiomyocyte impairment in HFpEF [10, 11]. Further, reflecting both inflammatory activity and macrophage-driven pro-fibrotic signaling, Galectin-3 has been associated with adverse outcomes in patients with overt HFpEF, while elevated levels are also frequently observed in individuals with T2DM [9]. Interestingly, IL1RL1 has demonstrated strong prognostic utility in acute coronary syndromes and both acute and chronic HF, rendering it a promosing marker to assess stress in mechanically overloaded cardiac myocytes [12].

Despite the diverse potential mechanistic backgrounds and presentations of HFpEF, many phenotypes share the underlying cardiac abnormalities as defined in current guidelines, for the assessment of which cardiovascular magnetic resonance (CMR) imaging has emerged as a first-line non-invasive diagnostic tool in cardiovascular disease, providing comprehensive insights on cardiac morphology, function, and tissue characterization, particularly in patients whose body morphology poses challenges to echocardiographic imaging [2, 13]. Leveraging the unique advantages of CMR, the present study aims to evaluate cardiac remodeling in obese and diabetic patients with HFpEF. Specifically, we will examine how hemodynamic, functional and structural changes on CMR present alongside with selected biomarkers with complementary roles in fibrotic, vascular, and inflammatory pathways in these patients and compare these findings with obese patients with T2DM and obese controls without cardiovascular disease (CVD) or comorbidities [10–12, 14]. By doing so, we hope to improve our understanding of the HFpEF pathophysiology and its differentiation from related comorbidities, ultimately contributing to more effective diagnostic strategies.

## Methods

### Study population

This was a prospective cross-sectional, observational study conducted at a German tertiary-care center between August 2018 and December 2020 and included a total of 35 obese subjects: 16 patients with T2DM, 13 patients with T2DM and HFpEF, and 6 healthy controls.

To be eligible, all patients had to be of the age of 40–80 years, have a BMI 25–40 kg/m2, and LVEF ≥ 50% by 2D echocardiography within six months prior to screening. Subjects with type 2 diabetes mellitus (T2DM) had a documented diagnosis and were on active antidiabetic treatment. Patients were included in the T2DM with HFpEF subgroup, if they presented with a history of signs and/or symptoms of heart failure according to the New York Heart Association Functional Classification (NYHA Class II–III) and all of the following were present: E/e’ratio ≥ 8 by 2D echo within the last 6 months, a total BNP > 220 pg/mL (> 26 pmol/L) or BNP ≥ 200 pg/mL (≥ 57.8 pmol/L) at time of screening, and active treatment with minimum one agent of HF guideline directed medical therapy. Obese controls had to be in good health based on medical records, labs, and vitals within normal ranges at time of screening. All participants were clinically stable with no medication changes within two weeks prior to inclusion. Subjects were excluded if they presented with a history of HFrEF or other specific cardiac diseases, such as non-diabetic cardiomyopathy, valve or pericardial disease, renal impairment (GFR < 30 ml/min/1.73m^2^), when they were unable to provide informed consent or had any MRI-specific contraindications.

For consistency, subgroups of patients are referred to as follows throughout the article: Patients who were overweight or obese and otherwise healthy are referred to as ‘obese controls without cardiovascular disease (CVD)’. Patients who were overweight or obese and had T2DM are referred to as ‘T2DM’, and overweight or obese patients with HFpEF and T2DM are referred to as ‘HFpEF with T2DM’.

Patients and controls underwent a thorough baseline clinical assessment including a detailed medical history, physical exam, 12-lead-ECG, and vitals, which was then followed by three consecutive study visits over a course of 21 days with repeat CMR scans, routine blood draws and biomarker tests.

This study adhered to the Declaration of Helsinki and was approved by the institutional ethics committee (EA2/083/18). All subjects provided written informed consent.

### CMR protocol

**Fig. 1:**
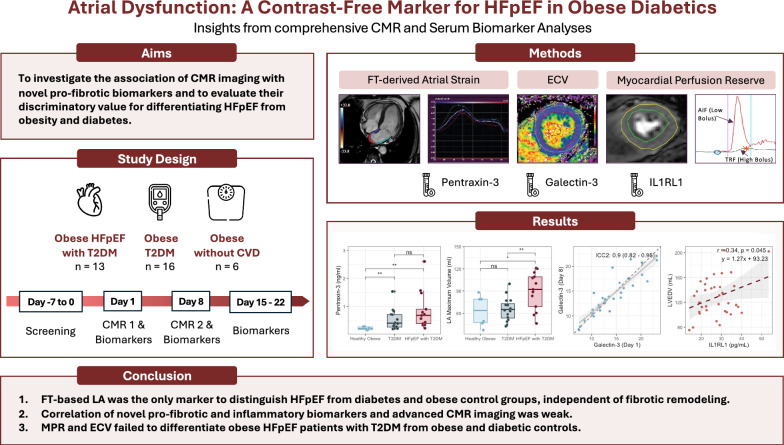
CMR, Cardiac Magnetic Resonance; ECV, Extracellular volume; FT, Feature tacking; HFpEF, Heart failure with preserved ejection fraction; IL1RL1, Interleukin-1 Receptor-Like 1; LA, Left atrium; LVEDV, Left ventricular end-diastolic volume; MPR, Myocardial perfusion reserve; Ns, not significant; T2DM, Type 2 diabetes mellitus

Study participants underwent a comprehensive CMR exam at study day 1, which was repeated after 7 days (as depicted in Fig. 1). In the initial phase of this study, up to 12 subjects (approximately six per cohort of T2DM patients and HFpEF with T2DM patients) underwent two CMR scans to evaluate myocardial perfusion reserve (MPR) and extracellular volume fraction (ECV). These scans served as a test–retest protocol to assess the variability of these parameters.

For the remaining study participants, two CMR scans were also performed. However, in addition to assessments of cardiac morphology and function in each scan, the first scan included ECV, while the second assessed MPR.

Scans were obtained complying with SCMR guidelines using a 3 T CMR scanner (Ingenia, Philips Healthcare, Best, The Netherlands), equipped with both anterior and built-in posterior coil arrays, employing up to 30 coil elements [15].

In brief, cine images were acquired in cardiac short-axis, two-, three- and four chamber view orientations using a retrospectively gated cine-CMR using a steady-state free precession (SSFP) sequence. Cine acquisition parameters are detailed in the Supplementary Marterial S1.

Following cine imaging, quantitative assessment of the myocardial perfusion reserve was conducted using the dual-bolus protocol as described by Ishida et al. [16] Adenosine infusion was initiated at a rate of 140 µg/kg/min and increased to 210 µg/kg/min in case of insufficient hemodynamic response, defined as a heart rate increase ≥ 10%. After approximately 3 min, a pre-bolus of 0.00375 mmol/kg of gadolinium-based contrast agent (Gadobutrol 1.0 mmol/mL, Gadovist®, Bayer AG, Leverkusen, Germany) was administered at a rate of 4 ml/s, followed by a 0.9% saline flush of 30 ml at a rate of 4 ml/s. After 10 dynamic measurements, a bolus of 0.0375 mmol/kg of gadolinium was injected at the same rate (4 ml/s), followed followed by a 0.9% saline flush of 30 ml at a rate of 4 ml/s, during breath-hold to minimize motion artifacts. Subsequently, adenosine infusion was discontinued, and following a 15 min recovery period, the same protocol was repeated for rest perfusion imaging. Data were acquired in real-time during the first pass of gadolinium. Peak-systolic, ECG-triggered (trigger delay = 110 ms), T1-weighted images were obtained using a spoiled gradient echo sequence (T1-TFE). Three LV short axis slices (base, mid and apex) were obtained corresponding to the American Heart Association 16-segment model. Saturation recovery preparation (saturation delay = 90 ms, no shared prepulse) was conducted for each slice for additional T1 weighting and reproducible contrast between slices. Typical imaging parameters were: field of view (FOV) RL/AP/FH = 250 mm/250 mm/42 mm, voxel size measured / reconstructed = 2.9 mm × 2.9 mm / 1.3 mm × 1.3 mm, slice thickness 8 mm, TR/TE = 2.6 ms/1.2 ms, flip angle = 15°, TFE shot duration per slice = 150 ms, parallel imaging SENSE = 2.3. Rest perfusion was considered abnormal if upon gadolinium first pass, a low signal on T1-weighted images was detected in a myocardial segment, persisting for a period of at least five heartbeats.

Finally, patients received a bolus of 0.0675 mmol/kg of gadolinium-based contrast agent to reach a full dose of 0.15 mmol/kg (2*0.0375 mmol/kg + 2*0.00375 mmol/kg + 0.0675 mmol/kg). Native and 15 min post contrast T1-mapping were performed using a modified Look-Locker (MOLLI) 5 s(3 s)3 s-scheme.

### CMR image analysis

Following imaging acquisition, de-identified images were sent to readers for blinded analysis. All acquired images were analyzed offline by experienced CMR investigators in accordance with current SCMR consensus recommendations for the standardized image interpretation and post processing in CMR using dedicated software (Medis Suite RE 4.0, Medis Medical Imaging Systems B.V., Leiden, The Netherlands) [17].

Left ventricular function and volumes were quantified as per the recommendation of the SCMR in a whole short axis (SAX) cine stack [17]. Endo- and epicardial borders were manually contoured in the end-diastolic and end-systolic phase with papillary muscles included in the left ventricular volumes [17].

For fibrosis quantification, the septal T1 relaxation time and extracellular volume (ECV) was calculated, for which regions of interest were conservatively drawn in the intraventricular septum, as well as in the left-ventricular blood pool on the basal slice in short axis view on both native and the corresponding post-contrast T1 mapping images [18]. ECV was calculated with the established equation utilizing native and post-contrast T1 relaxation times in conjunction with HCT values collected within 24 h of the CMR:

1 $$\text{ECV }= \left(1-\text{HCT}\right)\times \frac{\frac{1}{\text{T}1\text{myo post}}- \frac{1}{\text{T}1\text{myo native}})}{(\frac{1}{\text{T}1\text{blood post}}-\frac{1}{\text{T}1\text{blood native}} )}$$

Left atrial volumetric and strain parameters were determined using the QStrain function in Medis Suite (Medis Suite RE 4.0, Medis Medical Imaging Systems B.V., Leiden, The Netherlands) and averaged from segmentation of 2- and 4-chamber view cine images at end-diastolic and end-systolic frames, which were identified by the closure of the respective valve. Experienced investigators outlined the left atrial borders on the cine images, whilst carefully excluding adjacent structures and the pulmonary vein openings. Left atrial volumes and strain were recorded separately for each study visit.

For analysis of the perfusion images, an in-house developed MATLAB graphical user interface (GUI)-based interactive software package was employed based on the methods outlined in [19, 20]. It incorporated the described dual-bolus model-based deconvolution approach to estimate the tissue impulse response function to the measured arterial input function during the first-pass phase that then produces the measured total tissue response function. The myocardial blood flow was computed from the initial value of the estimated tissue impulse response function. The tissue impulse response function was modeled as a Fermi function R_F_(t) expressed as:

2 $${R}_{F}(t)=\frac{A}{[\text{exp}\left[\frac{t-\upmu }{k}\right]+1}$$

The shape parameters of the Fermi function *A*, *μ* and *k* were estimated through a non-linear least squares Levenberg–Marquardt optimization algorithm.

The software first took as input the first pass images corresponding to the low bolus input, the first pass images corresponding to the high bolus input and the scaling factor that was the ratio of the concentrations of the two boluses. Second, the GUI based algorithm enabled manual point-to-spline-based segmentation of the blood pool used to extract the scaled temporal curve for the arterial input function from the first pass low bolus images. Third, utilizing the GUI, manual segmentation of the myocardial region was performed to generate the temporal curve for the total tissue response function from the first pass high bolus images. Finally, the portion of the curves corresponding to the first pass temporal region only was extracted, smoothed, registered temporally and then used for the Fermi model-based deconvolution approach to generate the impulse response Fermi curve from which the myocardial blood flow value was calculated.

### Serum biomarkers

Galectin-3 was measured in serum using an ELISA kit (BG Medicine Catalog #12,836, Foxboro, Massachusetts, USA) at Q2 solutions. The assay was validated with an intra-assay CV of 0.9–3.5% and inter-assay CV of 2.0–4.4%. The reportable range of the Galectin-3 assay is 3.13 ng/mL to 100 ng/mL in human serum.

Pentraxin 3 was measured in K2EDTA plasma using a Quantikine ELISA kit (RD Systems Cat #DPTX30B, Minneapolis, Minnesota, USA) at Merck Co., Inc., Rahway, NJ, USA. This assay was validated with an intra-assay CV of 1.5–2.5% and inter-assay CV of 5.3–7.3%. The reportable range of the pentraxin 3 assay is 0.094 ng/mL to 192 ng/mL in human K2EDTA plasma.

Interleukin-1 Receptor-Like 1 (IL1RL1, also known as soluble suppression of tumorigenicity 2 or sST2) was measured in serum using the Presage ST2 ELISA kit (Critical Diagnostics, San Diego, California, USA) at Q2 solutions. The assay was validated with an intra-assay CV of 2.1–4.9% and inter-assay CV of 5.2–7.6%. The reportable range of the IL1RL1 assay is 1.6 ng/mL to 200 ng/mL in human serum.

### Statistical analysis

Continuous data are presented as mean ± standard deviation (SD) with differences between subgroups compared using one-way analysis of variance (ANOVA) and Tukey test for post hoc comparison, as appropriate. Categorical data are presented as numbers and percentages and were compared between subgroups using Pearson’s Chi-squared test. For parameters with measurements available for two or more visits, the values of all measurements were averaged for further analyses. Associations between imaging indices and circulating biomarkers were evaluated using Pearson correlation analysis. To determine the impact of individual parameters on the probability of HFpEF, univariable logistic regression was performed. Subsequently, variables with significant associations were further analyzed in multivariable logistic regression models, additionally adjusting for age, sex, and body mass index (BMI). For both regression and correlation analyses, skewed parameters (i.e., Pentraxin-3) were log-transformed to improve normality. Receiver operating characteristic (ROC) analysis was conducted to evaluate the diagnostic performance of imaging and serum biomarker variables in distinguishing HFpEF from non-HFpEF participants. For each parameter, ROC curves were generated using the pROC package in R, and the area under the curve (AUC) with 95% confidence intervals was calculated. Optimal thresholds were determined using Youden’s index, and corresponding sensitivity, specificity, positive and negative predictive values, and accuracy were extracted. Reproducibility of these parameters was assessed using measurements from days 1 and 8, aligning with the timing of CMR acquisitions. Intra-class correlation coefficients (ICC, more specifically ICC2) were calculated following Cicchetti’s guidelines, employing the irr package in R [21]. Sensitivity analyses excluding any patients with medical history positive for any type of atrial arrhythmia were performed and are presented as Supplementary Material S4.

Originally, this study was designed with posterior probability threshold set at 80%, targeting a sample size of 20 patients per group. However, for this article, we conducted exploratory analyses using conventional statistical significance thresholds (two-sided *p*-value < 0.05), which differ from the prespecified power calculation. All statistical analyses were performed using R version 4.3.0 (The R Foundation for Statistical Computing, Vienna, Austria).

## Results

### Patient characteristics

The clinical characteristics of the study population are presented in Table 1. The cohort included 35 subjects (23% female, n = 8) with a mean age of 64 ± 8 years and a mean BMI of 30.7 ± 3.9 kg/m2, the latter of which differed significantly among the groups (*p* = 0.046). Patients with HFpEF and T2DM tended to be slightly older and have a higher prevalence of cardiovascular comorbidities than patients in the other subgroups. However, a statistically significant difference was only observed for the presence of arterial hypertension (*p* < 0.001). All studied subjects had hematocrit levels within normal limits. Patients with a history of T2DM (regardless, whether with or without HFpEF) exhibited significantly higher hemoglobin A1c levels compared to obese controls without CVD (*p* = 0.026). NT-proBNP was within normal limits in the T2DM cohort and obese controls without CVD but significantly elevated in the HFpEF with T2DM group (*p* < 0.001).

**Table 1:** Patient characteristics

Variable	*n*	Obese without CVD *n* = 6^1^	T2DM *n* = 16^1^	HFpEF with T2DM *n* = 13^1^	*p*-value^*2*^
*Demographics*					
Age (years)	35	62 ± 2	61 ± 11	68 ± 4	0.115
Female	35	2 (33%)	1 (6.3%)	5 (38%)	0.097
Body Mass Index (kg/m2)	35	27.7 ± 1.3	30.6 ± 3.1	32.3 ± 4.7	**0.046**
*Comorbidities*					
Arterial Hypertension	35	0 (0%)	12 (75%)	13 (100%)	** < 0.001**
Hypercholesterolemia	35	0 (0%)	3 (19%)	2 (15%)	0.529
Diabetic Burden (years)^3^	28	NA	7.9 ± 6.4	11.1 ± 10.8	0.346
Atrial Fibrillation	35	0 (0%)	0 (0%)	2 (15%)	0.166
Atrial Ectopic Tachycardia	35	0 (0%)	0 (0%)	1 (7.7%)	0.419
*Medication*					
ACE inhibitors	35	0 (0%)	5 (31%)	4 (31%)	0.285
Angiotensin II Receptor Blockers	35	0 (0%)	7 (44%)	8 (62%)	**0.042**
Beta Blockers	35	0 (0%)	3 (19%)	7 (54%)	**0.027**
Calcium Channel Blockers	35	0 (0%)	7 (44%)	5 (38%)	0.145
SGLT2 Inhibitors	35	0 (0%)	5 (31%)	3 (23%)	0.299
GLP-1 Receptor Agonists	35	0 (0%)	4 (25%)	1 (7.7%)	0.277
Oral antidiabetics	35	0 (0%)	16 (100%)	13 (100%)	** < 0.001**
Insulins	35	0 (0%)	3 (19%)	1 (7.7%)	0.406
Statins	35	0 (0%)	8 (50%)	5 (38%)	0.096
Other lipid-lowering agents	35	0 (0%)	2 (13%)	2 (15%)	0.609
Diuretics	35	0 (0%)	5 (31%)	4 (31%)	0.285
Anticoagulants	35	0 (0%)	0 (0%)	3 (23%)	0.062
Antiplatelets	35	0 (0%)	4 (25%)	3 (23%)	0.401
*Labs*					
Hemoglobin (g/dL)	35	14.53 ± 1.17	14.46 ± 1.13	13.66 ± 1.19	0.144
Total Protein (g/dL)	35	7.15 ± 0.40	7.31 ± 0.28	7.16 ± 0.39	0.421
Creatinine (mg/dL)	35	0.87 ± 0.14	0.99 ± 0.21	1.03 ± 0.25	0.304
Hemoglobin A1c (%)	18	5.23 ± 0.29	6.78 ± 1.69	6.77 ± 0.55	**0.026**
NT-proBNP (pmol/L)	34	6 ± 2	6 ± 3	48 ± 23	** < 0.001**
*Cardiac Magnetic Resonance*					
LVEDV (mL)	35	133 ± 27	127 ± 30	127 ± 36	0.913
LVSV (mL)	35	79 ± 9	75 ± 23	77 ± 17	0.919
LVEF (%)	35	61 ± 9	59 ± 9	62 ± 8	0.672

^1^Mean ± SD; n (%)

^2^One-way ANOVA; Pearson’s Chi-squared test

^3^Year of Onset of T2DM unknwon for one patient in the HFpEF with T2DM subgroup

With regard to left ventricular morphology and volumetrics, no significant differences were observed in parameters such as left ventricular end-diastolic volume (LVEDV, *p* = 0.913), left ventricular stroke volume (LVSV, *p* = 0.919) or left ventricular ejection fraction (LVEF, *p* = 0.672).

### Microvascular dysfunction and diffuse fibrosis

**Fig. 2:**
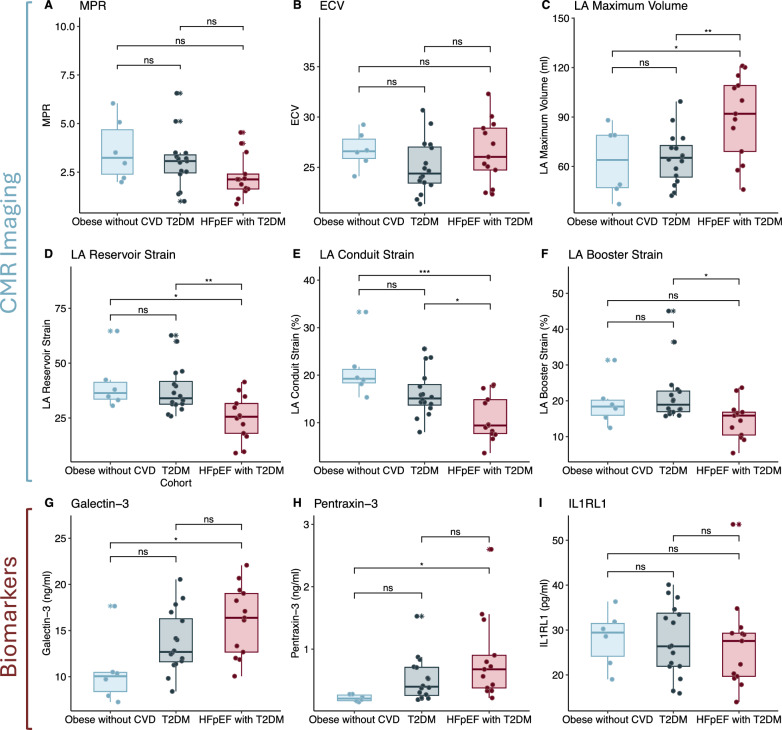
Group comparisons of advanced CMR imaging metrics **A**–**F** and pro-inflammatory serum biomarkers **G**–**I** were conducted for obese controls without CVD (sky blue), obese with type 2 diabetes (T2DM, dark gray), and HFpEF with T2DM (red) subgroups using Tukey’s test. Statistical significance was defined as *P* < 0.05. CMR, Cardiac Magnetic Resonance; ECV, Extracellular volume; HFpEF, Heart failure with preserved ejection fraction; IL1RL1, Interleukin-1 Receptor-Like 1; LA, Left atrium; MPR, Myocardial perfusion reserve; Ns, not significant; Obese without CVD, Obese controls without cardiovascular disease; T2DM, Type 2 diabetes mellitus

Detailed findings of mean values ± SD and of the ANOVA are outlined in Table 2, while the corresponding post hoc Tukey tests are visualized in Fig. 2. MPR was available for 34 patients (one patient in the T2DM group was not included in the analysis due to unreadable results), with the highest values in the obese without CVD group (3.63 ± 1.62) and the lowest values observed in HFpEF with T2DM (2.27 ± 1.10), although there were no statistically significant differences between any of the groups (ANOVA, *p* = 0.108).

**Table 2:** Findings

Variable	n	Obese without CVD n = 6^1^	T2DM n = 16^1^	HFpEF with T2DM n = 13^1^	*p*-value^2^
*Perfusion & Fibrosis*					
MPR	34	3.63 ± 1.62	3.05 ± 1.41	2.27 ± 1.10	0.108
ECV (%)	35	26.73 ± 1.81	25.03 ± 2.65	26.54 ± 3.14	0.251
*Atrial Morphology & Function*					
Minimum LA Volume (mL)	35	35 ± 17	37 ± 13	64 ± 21	** < 0.001**
Maximum LA Volume (mL)	35	63 ± 21	65 ± 16	90 ± 25	**0.005**
LA Reservoir Strain (%)	35	41 ± 12	38 ± 11	25 ± 10	**0.005**
LA Conduit Strain (%)	35	21.2 ± 6.3	16.1 ± 4.8	11.0 ± 4.8	**0.001**
LA Booster Strain (%)	35	19 ± 7	22 ± 8	15 ± 5	**0.032**
*Serum Biomarkers*					
Galectin-3 (ng/mL)	35	10.6 ± 3.7	13.8 ± 3.3	16.1 ± 3.8	**0.014**
Pentraxin-3 (ng/mL)	35	0.21 ± 0.05	0.52 ± 0.35	0.84 ± 0.67	**0.031**
Interleukin-1 Receptor-Like 1 (pg/mL)	35	28 ± 6	28 ± 8	27 ± 10	0.928

^1^Mean ± SD

^2^One-way ANOVA

Extracellular volume (ECV) measurements were within normal limits across all groups, ranging from 25.03% ± 2.65% in the T2DM group to 26.73% ± 1.81% in the obese controls without CVD, with differences between the groups not reaching statistical significance (ANOVA, *p* = 0.251).

### Left atrial morphology and function

Left atrial (LA) morphology and function demonstrated substantial differences, particularly in the HFpEF with T2DM group. Minimum LA volumes were significantly larger in HFpEF with T2DM (64 ± 21 mL) compared to obese controls without CVD (35 ± 17 mL, *p* = 0.004) and T2DM (37 ± 13 mL, *p* < 0.001). Similarly, maximum LA volumes were significantly larger in HFpEF with T2DM (90 ± 25 mL) compared to obese controls without CVD (63 ± 21 mL, *p* = 0.032) and T2DM (65 ± 16 mL, *p* = 0.008). There were no statistically significant differences for either of the parameters between the obese controls without CVD and T2DM group (*p* = 0.975).

Moreover, LA reservoir strain was consistently reduced in HFpEF with T2DM (25 ± 10%) compared to both obese controls without CVD (41 ± 12%, *p* = 0.019) and T2DM (38 ± 11%, *p* = 0.010), while no significant differences in LA reservoir strain values were found between obese controls without CVD and patients with T2DM (*p* = 0.862). This effect was also observed for LA conduit strain, with significantly lower values in HFpEF with T2DM (11.0 ± 4.8%) compared to obese controls without CVD (21.2 ± 6.3%, *p* = 0.001) and T2DM (16.1 ± 4.8%, *p* = 0.030). However, no significant differences were observed between obese controls without CVD and the T2DM group (*p* = 0.108).

Regarding LA booster strain, patients with HFpEF and T2DM exhibited lower values (15 ± 5%) compared to T2DM (22 ± 8%, 0.025). However, the difference between HFpEF with T2DM and obese controls without CVD (19 ± 7%) was not statistically significant (*p* = 0.357), neither was the difference between obese controls without CVD and patients with T2DM (*p* = 0.757).

### Plasma biomarker levels

Galectin-3 levels were 10.6 ± 3.7 ng/mL in obese controls without CVD and 13.8 ± 3.3 ng/mL in T2DM patients (*p* = 0.164 for obese controls without CVD vs. T2DM), and highest in HFpEF patients with T2DM (16.1 ± 3.8 ng/mL), the only significant difference of which was reached when HFpEF with T2DM was compared to obese controls without CVD (*p* = 0.011, with *p* = 0.226 for HFpEF with T2DM vs. T2DM). Similarly, Pentraxin-3 levels were significantly lower in obese controls without CVD (0.21 ± 0.05 ng/mL) than in HFpEF patients with T2DM (0.84 ± 0.67 ng/mL, *p* = 0.031), but not than patients with T2DM (0.52 ± 0.35 ng/mL, *p* = 0.391), while levels between the two latter groups were also not significantly different (*p* = 0.176). In contrast to Pentraxin-3 and Galectin 3, Interleukin-1 Receptor-Like 1 levels did not show a significant differences across the groups, with levels of 28 ± 6 pg/mL in obese controls without CVD, 28 ± 8 pg/mL in T2DM patients, and 27 ± 10 pg/mL in HFpEF patients with T2DM (ANOVA, *p* = 0.928).

### Correlation of imaging and plasma biomarkers

In the overall study population, log-transformed Pentraxin-3 demonstrated a negative correlation with LA conduit strain values (*r* = − 0.35, *p* = 0.04). Galectin-3 was negatively correlated with LVESV (*r* = − 0.39, *p* = 0.02), while IL1RL1 exhibited a mild positive correlation with LVESV (*r* = 0.39, *p* = 0.021) and LVEDV (*r* = 0.34, *p* = 0.045). Within the subgroup of patients with HFpEF and T2DM, IL1RL1 levels were positively associated with increasing LA minimal volume (*r* = 0.57, *p* = 0.041) and with both end-diastolic (*r* = 0.69, *p* = 0.009) and end-systolic (*r* = 0.75, *p* = 0.003) volumes. Additionally, IL1RL1 showed a negative correlation with LA function, as indicated by LA booster strain (*r* = − 0.57, *p* = 0.043). Scatter plots, including the corresponding Pearson’s correlation coefficients and regression equations, are presented in Supplementary Material S2.

### Logistic regression analyses for HFpEF

In univariable logistic regression (Table 3), Galectin-3 and Pentraxin-3 emerged as significant serum biomarkers associated with HFpEF as a dichotomous outcome. Specifically, Galectin-3 was positively associated with an odds ratio (OR) of 1.25 (95% CI 1.04–1.57, *p* = 0.029), while Pentraxin-3 demonstrated an OR of 4.03 (95% CI 1.37–15.4, *p* = 0.021). In contrast, IL1RL1 did not show a significant association (OR 0.98, 95% CI 0.90–1.07, *p* = 0.697).

**Table 3:** Logistic regression analyses

Variable	Univariable logistic regression		Multivariable Logistic Regression*	
	OR (95% CI)	*p*-value	OR (95% CI)	*p*-value
*Hemodynamics & Fibrosis*				
ECV (%)	1.15 (0.90, 1.51)	0.279	–	–
MPR	0.53 (0.24, 0.97)	0.069	–	–
*LV and LA Function*				
LV EDV (mL)	1.00 (0.97, 1.02)	0.848	–	–
LA Max Vol (mL)	1.06 (1.02, 1.11)	**0.006**	1.13 (1.05, 1.28)	**0.011**
LA Reservoir Strain (%)	0.84 (0.71, 0.94)	**0.011**	0.71 (0.44, 0.89)	**0.039**
LA Conduit Strain (%)	0.76 (0.60, 0.90)	**0.006**	0.57 (0.32, 0.82)	**0.018**
LA Booster Strain (%)	0.78 (0.60, 0.93)	**0.023**	0.70 (0.48, 0.89)	**0.019**
*Serum Biomarkers*				
Galectin-3 (ng/mL)	1.25 (1.04, 1.57)	**0.029**	1.17 (0.94, 1.52)	0.183
Pentraxin-3 (ng/mL)^†^	4.03 (1.37, 15.4)	**0.021**	3.34 (0.92, 15.7)-	0.085
IL1RL1 (pg/mL)	0.98 (0.90, 1.07)	0.697	–	–

*p*-values < 0.05 are highlighted in bold

*Included the respective variable significant in univariable logistic regression and additionally accounted for age, sex and BMI

^†^Values for log-transformed Pentraxin-3

Among imaging and hemodynamic markers, neither LVEDV (OR 1.00, 95% CI 0.97–1.02, *p* = 0.848) nor LVEF (OR 1.03, 95% CI 0.95–1.13, *p* = 0.427) showed significant association with HFpEF. Neither MPR nor ECV were statistically predictive, with an OR of 0.53 (95% CI 0.24–0.97, *p* = 0.069) and OR 1.15, 95% CI 0.90–1.51, *p* = 0.279), respectively. Atrial parameters, however, emerged as significant discriminators in the univariable model. Specifically, higher LA maximum volume was significantly associated with HFpEF (OR 1.06, 95% CI 1.02–1.11, *p* = 0.006), as were reductions in LA reservoir (OR 0.84, 95% CI 0.71–0.94, *p* = 0.011), conduit (OR 0.76, 95% CI 0.60–0.90, *p* = 0.006), and booster strain (OR 0.78, 95% CI 0.60–0.93, *p* = 0.023).

For variables significant in univariable logistic regression, multivariable logistic regression adjusted for age, sex, and BMI was performed (Table 3). Among serum biomarkers, the association of Galectin-3 (OR 1.17, 95% CI 0.94–1.52, *p* = 0.183) and Pentraxin-3 (OR 3.34, 95% CI 0.92–15.7, *p* = 0.085) with HFpEF weakened and failed to reach significance in the adjusted model. In contrast, functional parameters of LA strain maintained their independent predictive value. Increases in LA reservoir strain were associated with lower odds for HFpEF, with an OR of 0.72 (95% CI 0.44–0.89, *p* = 0.039), while LA conduit strain demonstrated an OR of 0.57 (95% CI 0.32–0.82, *p* = 0.018), and LA booster strain an OR of 0.70 (95% CI 0.48–0.89, *p* = 0.019). LA maximum volume was positively associated with HFpEF in the adjusted model, with an OR of 1.13 (95% CI 1.05–1.28, *p* = 0.011).

### ROC-analysis

In ROC analyses, left atrial functional parameters demonstrated the highest discriminatory ability, whereas markers of diffuse fibrosis and microvascular dysfunction, as well as serum biomarkers, yielded lower AUCs and more variable performance. A vizualisation and summary of the ROC-derived diagnostic metrics are provided in Supplementary Material S3.

### Inter-scan reproducibility

**Fig. 3:**
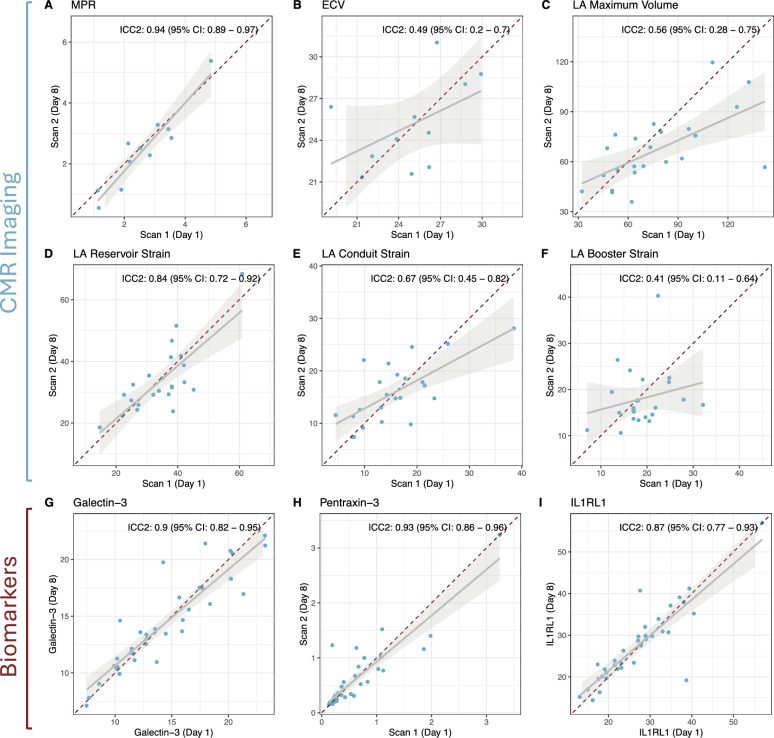
Reproducibility of of advanced CMR imaging metrics **A**–**F** and pro-inflammatory serum biomarkers **G**–**I** from days 1 and 8, for when to measurements were acquired. Intra-class correlation coefficients (ICC2) were calculated following Cicchetti’s guidelines and are indicated in the respective graph ECV, Extracellular volume; CMR, Cardiac Magnetic Resonance; ECV, Extracellular volume; HFpEF, Heart failure with preserved ejection fraction; ICC, Intra-class correlation coefficient; IL1RL1, Interleukin-1 Receptor-Like 1; LA, Left atrium; MPR, Myocardial perfusion reserve; Ns, not significant; T2DM, Type 2 diabetes mellitus

A total of 11 patients (*n* = 6 in the T2DM group and *n* = 5 in the HFpEF with T2DM group) had repeated analyses for MPR. Inter-study repeatability showed an excellent ICC(2) of 0.94 (95% CI 0.89–0.97, Fig. 3). In contrast, inter-study repeatability (*n* = 11) for ECV showed only a moderate ICC(2) of 0.49 (95% CI 0.2–0.7).

In 23 patients (*n* = 7 for Obese HFpEF with T2DM, *n* = 10 for Obese with T2DM, *n* = 6 for obese controls without CVD), repeat measurements for left atrial volumetry and function were performed to assess reproducibility (Fig. 3). LA maximal volume demonstrated good reproducibility with ICC of 0.56 (95% CI 0.28–0.75) and LA minimal volume exhibited an ICC of 0.41 (95% CI 0.11–0.65, not shown). LA reservoir strain (ICC = 0.84, 95% CI 0.72–0.92) and LA conduit strain (ICC = 0.67, 95% CI 0.45–0.82) showed good reproducibility. LA booster strain, however, showed much greater variability and ICC revealed moderate agreement of only 0.41 (95% CI 0.11–0.64) in between the two measurements.

Reproducibility of all three serum biomarkers (reproducibility assessed for the two CMR visits) showed excellent ICCs (Galectin-3: 0.9, 95% CI 0.82–0.95; Pentraxin-3: 0.93, 95% CI 0.86–0.96; IL1RL1: 0.87, 95% CI 0.77–0.93).

## Discussion

This exploratory study provides a comprehensive assessment of cardiac structure, function, tissue composition, and hemodynamics, utilizing both native and contrast-enhanced non-invasive CMR imaging in conjunction with serum biomarker analyses in obese patients with HFpEF and T2DM. By comparing these patients to control groups with similar comorbidities, we aimed to identify subtle but clinically relevant differences distinguishing individuals with clinically manifest HFpEF from those with related, potentially contributory conditions.

### Advanced CMR imaging

Our analyses identified diminished atrial function as the only independent marker capable of distinguishing HFpEF patients from the other subgroups after adjustment for age, sex, and BMI in a multivariable model. Specifically, HFpEF patients with T2DM presented with significantly reduced LA reservoir, conduit and booster strain values in feature tracking CMR. The clinical utility and diagnostic incremental value of LA strain, especially in patients with preserved ejection fraction and early diastolic dysfunction have been demonstrated [22, 23]. In addition to this, prior studies have shown impaired LA function to be associated with exercise intolerance, elevated risk for atrial arrhythmia and increased mortality in HFpEF patients [24–27]. For instance, Chirinos et al. demonstrated an association with higher incidence of HF hospitalization or death for both impaired LA reservoir strain and conduit strain in patients with HF regardless of ejection fraction, and Melenovsky et al. found higher mortality rates in a HFpEF subgroup with decreased atrial function [25, 26]. Reddy et al. reported an increased risk for new onset or progression of atrial fibrillation among HFpEF patients with impaired LA reservoir strain, with 65% of their cohort showing no history of atrial fibrillation [24]. In our study, only 3 out of the 35 participants (8.6%) had a history of atrial arrhythmia of any kind, and sensitivity analyses showed similar results when these three patients were excluded, raising the question whether patients without a documented history of atrial arrhythmia may have undiagnosed subclinical arrhythmic disease contributing to symptom severity in this cohort. Depite this, ROC analyses (Supplementary Material S3) reinforced these observations with regards to the superior diagnostic performance of LA strain parameters relative to other imaging and biomarker variables tested in this cohort. While the full prognostic value of LA impairment in HFpEF is yet to be uncovered, first studies have shown that certain interventions may have a favorable effect on LA function, particularly in populations with T2DM and obesity. In a recent CMR-based study, a low-energy meal replacement plan (~ 810 kcal/day) was found to be more effective than aerobic exercise in improving LA reservoir and booster strain, possibly mediated by significant reductions in BMI [28]. Moreover, several studies in diabetic patients with or without HFmrEF or HFpEF have demonstrated that the addition of SGLT2 inhibitors to existing guideline-directed medical therapy led to better glycemic control and improvement of diastolic measures as well as LA functions [29–31].

Surprisingly, we could not reproduce the marked microvascular dysfunction reported in other HFpEF studies [32, 33]. In fact, the difference in between the HFpEF with T2DM group and the other subgroups did not reach statistical significance, with mean MPR values across all subgroups exceeding values observed by Arnold et al., who also demonstrated the prognostic relevance of reduced MPR in HFpEF patients [33]. Microvascular dysfunction is a known feature in both patients with diabetes and obesity, which may have mitigated the observed differences between groups [34, 35]. With mean values continuously declining with increasing number of comorbidities, it seems plausible that coronary microvascular dysfunction may gradually contribute to the HFpEF syndrome– yet, there was notable overlap between the groups and it remains unclear whether a larger sample size might have rendered a statistically significant difference in this cohort.

ECV values were similar across all three groups, showing no specific trend. Interestingly, these findings align with Arnold et al., who also reported no association between MPR and CMR-based diffuse fibrosis markers (i.e., ECV or indexed ECV) [33]. While ECV has been proposed as a discriminating variable between HFpEF and other cardiac conditions (i.e. hypertensive heart disease), our data revealed no significant elevation in ECV among HFpEF patients compared with controls [36]. It is important to note that previous studies found increased ECV in T2DM patients, particularly those with vascular complications, whereas T2DM without complications seemed to be linked to lower ECV values [37, 38]. In our cohort, which was characterized by obesity and T2DM but had no history of peripheral vascular complications, the absence of significant ECV elevation may suggest an earlier stage of disease in which ECV may be “lowered” by the comorbidities while ECV-elevating fibrotic remodeling may not yet be as prominent. Further, it must be noted that inter-scan reproducibility of ECV in our study was surprisingly low, despite highly standardized image acquisition and post-processing, and as such, may have limited the interpretability of our results.

### Serum biomarkers reflecting inflammation and matrix remodeling

This hypothesis would further be supported by our biomarker analysis, which revealed inflammatory and pro-fibrotic markers largely within normal limits across all groups. Yet, Galectin-3 and Pentraxin-3 showed an upward trend in patients with T2DM, and especially in those with both T2DM and HFpEF, supporting the hypothesis by Paulus and Tschöpe that inflammatory and pro-fibrotic pathways may contribute to HFpEF progression in these patients [7]. Nonetheless, incremental changes in Pentraxin-3 were too modest to distinguish HFpEF from the other groups, and while Galectin-3 initially appeared linked to HFpEF in univariable linear regression, it lost statistical significance when adjusted for age, sex, and BMI. The correlation between these biomarkers and parameters assessed in CMR was rather weak and inconsistent, providing little direction. Unlike Galectin-3 and Pentraxin-3, IL1RL1 showed no significant differences across the subgroups but was the only biomarker that demonstrated a moderate correlation with ventricular volumes, as well as with atrial remodeling and function within the HFpEF subgroup. This observation aligns with previous studies that found IL1RL1 to be linked to impaired left atrial function and prognosis in HFpEF [8, 39].

However, these biomarkers cardiac specificity [40–42]. Fibrosis and vascular remodeling, particularly in patients with T2DM, often reflect a systemic net effect of inflammation, oxidative stress, and of matrix turnover and fibroblast remodeling across multiple organ systems, including the kidneys, liver, and brain. Consequently, advanced imaging modalities, such as CMR, may provide superior specificity in identifying and characterizing the cardiac endpoint of interest, including HFpEF. This highlights the potential of CMR as a more targeted tool for cardiac phenotyping in the context of multi-organ involvement.

Given the diverse clinical presentation of HFpEF and the limitations of existing diagnostic tools, there is an urgent need for accessible, clinically applicable markers to guide diagnostic workups. In this context, findings from our study suggest that early functional testing, such as FT-based LA strain analysis, could evolve into an effective diagnostic tool when assessing obese and diabetic patients for potential HFpEF. While our observations require validation in larger, prospectively designed cohorts, they highlight the potential utility that LA FT may offer for native, reproducible and also retrospective assessments in patients at risk for HFpEF, especially in comparison to ECV or MPR that may be less pronounced in earlier disease stages and require multi-step analyses, including contrast-administration and stress testing. With commercially available software and the continuously rising use of artificial intelligence for image post-processing, such analyses could easily be implemented into routine practice and trigger more targeted follow-ups—including invasive assessments if warranted.

### Limitations and future directions

We acknowledge several limitations of this study. First, it is important to note that the results should primarily be regarded as exploratory and hypothesis-generating, as the presented analyses deviated from the originally specified study protocol. Given that the final sample size was smaller than the originally planned *n* = 20 per group, the study may have been underpowered to detect certain differences. For instance, in ROC analyses, several LA-derived parameters demonstrated identical diagnostic performance at their respective optimal thresholds—while similar classification profiles across participants are not unexpected given the physiological interdependence of these measures, these findings may also, in part, reflect the limited sample size and therefore warrant validation in larger cohorts. Generally, small sample sizes may increase the risk of both type I and type II errors, thereby affecting the precision of estimated effect sizes and widening confidence intervals. Consequently, some of the statistically non-significant findings, particularly for previously established parameters such as MPR and ECV, may be attributed to the limited sample size, which necessitates larger effect sizes to achieve statistical significance. Moreover, by selecting control groups with obesity and T2DM, we intentionally introduced a bias toward the null hypothesis. Second, late gadolinium enhancement (LGE) quantification was not performed in this study, restricting our ability to assess focal fibrosis and myocardial scarring. While myocardial injury detectable by LGE has prognostic relevance, its occurrence is less frequent in HFpEF [30]. Third, Despite substantial efforts to ensure representative recruitment, only approximately one quarter of the herein presented study were women. We acknowledge that the underrepresentation of female patients may limit the generalizability of our findings. Future studies with balanced sex representation and larger sample size are needed to explore potential sex-specific differences in the context of HFpEF and T2DM. Lastly, NT-proBNP levels were utilized as an inclusion criterion for the HFpEF with T2DM subgroup, in alignment with current guidelines and scoring systems. As a result, NT-proBNP was excluded from subsequent regression models and analyses.

### Conclusion

In this observational cohort, we demonstrated that CMR markers of diffuse fibrosis and microvascular dysfunction may not effectively differentiate obese patients with HFpEF and diabetes from obese or diabetic controls. However, feature-tracking-based assessment of left atrial function demonstrated significantly lower values in HFpEF patients and may offer a practical, reproducible marker with potential value to distinguish HFpEF from associated comorbidities, independent of fibrotic remodeling. Given the exploratory nature and limited sample size, these findings require validation in larger, prospective studies.

## Supplementary Information

Supplementary material 1 (PDF 925 KB)

## Data Availability

The data underlying this article are available in the article and its online supplementary material, and additional data may be shared upon reasonable request to the corresponding author. Data will only be shared with academic institutions after signing a data sharing agreement.
