# Supplementary material for: Atrial dysfunction: a contrast-free marker for HFpEF in obese diabetics—insights from comprehensive CMR and serum biomarker analyses
Source: Cardiovasc Diabetol. 2025 Jun 18;24:258. doi: 10.1186/s12933-025-02808-3 (PMC12175437; doi:10.1186/s12933-025-02808-3)
Supplement: Supplementary file 1 — Supplementary material 1 (PDF 925 KB) [file 12933_2025_2808_MOESM1_ESM.pdf]

Supplementary Material

Atrial dysfunction as a contrast-free marker to identify HFpEF in obese diabetics: Insights from a comprehensive CMR and biomarker assessment

Table of Contents

S1. Imaging Parameters for strain analysis ... 2

S2. Pearson Correlation ... 3

2.1 Pearson's Correlation for Serum Biomarkers and CMR Imaging across all study subjects ... 3

2.2 Pearson's Correlation for Serum Biomarkers and CMR Imaging across all study subjects – continued. 4

2.3 Pearson's Correlation for Serum Biomarkers and CMR Imaging across HFpEF with T2DM cohort only 5

2.4 Pearson's Correlation for Serum Biomarkers and CMR Imaging across HFpEF with T2DM cohort only – continued ... 6

S3. Receiver Operating Curve Analysis ... 7

3.1 Visualization ... 7

3.2 Summary of Receiver Operating Analysis Statistics ... 8

S4. Sensitivity analysis for subgroup without atrial arrhythmia ... 9

4.1 Patient Characteristics for subgroup of patients without medical history for atrial arrhythmia ... 9

4.2 Findings for subgroup pf patients without medical history for atrial arrhythmia ... 11

4.3 Logistic Regression Analyses for subgroup of patients without medical history for atrial arrhythmia 12

4.4 Comparative analysis of Serum Biomarkers and CMR Imaging across Subgroup of patients without medical history for atrial arrhythmia ... 13

4.5 Pearson's Correlation for Serum Biomarkers and CMR Imaging across Subgroup of patients without medical history for atrial arrhythmia ... 14

4.6 Pearson's Correlation for Serum Biomarkers and CMR Imaging Subgroup of patients without medical history for atrial arrhythmia – continued ... 15

4.7 Pearson's Correlation for Serum Biomarkers and CMR Imaging across HFpEF with T2DM cohort without medical history for atrial arrhythmia only ... 16

4.8 Pearson's Correlation for Serum Biomarkers and CMR Imaging across HFpEF with T2DM cohort without medical history for atrial arrhythmia only – continued ... 17

4.9 Visualization of Receiver Operating Analysis across HFpEF with T2DM cohort without medical history for atrial arrhythmia only ... 18

4.10 Summary of Receiver Operating Analysis Statistics across HFpEF with T2DM cohort without medical history for atrial arrhythmia only ... 19

S1. Imaging Parameters for strain analysis

LA volumes and strain were derived from cine CMR images acquired in two-, three-, and four-chamber orientations using a standard retrospectively gated steady-state free precession (SSFP) sequence with breath-holding at end-expiration. The following imaging parameters were used: temporal resolution <35 ms with minimum 30 heart phases, slice thickness 6 mm, inter-slice gap 0 mm, flip angle 45°, acquired voxel size ~1.9 × 1.9 mm (reconstructed: 1.1 × 1.1 mm).

Image post-processing and strain analysis were performed using commercially available software (Medis Suite, Version RE 4.0, Medis Medical Imaging, Leiden, The Netherlands).

S2. Pearson Correlation

2.1 Pearson's Correlation for Serum Biomarkers and CMR Imaging across all study subjects

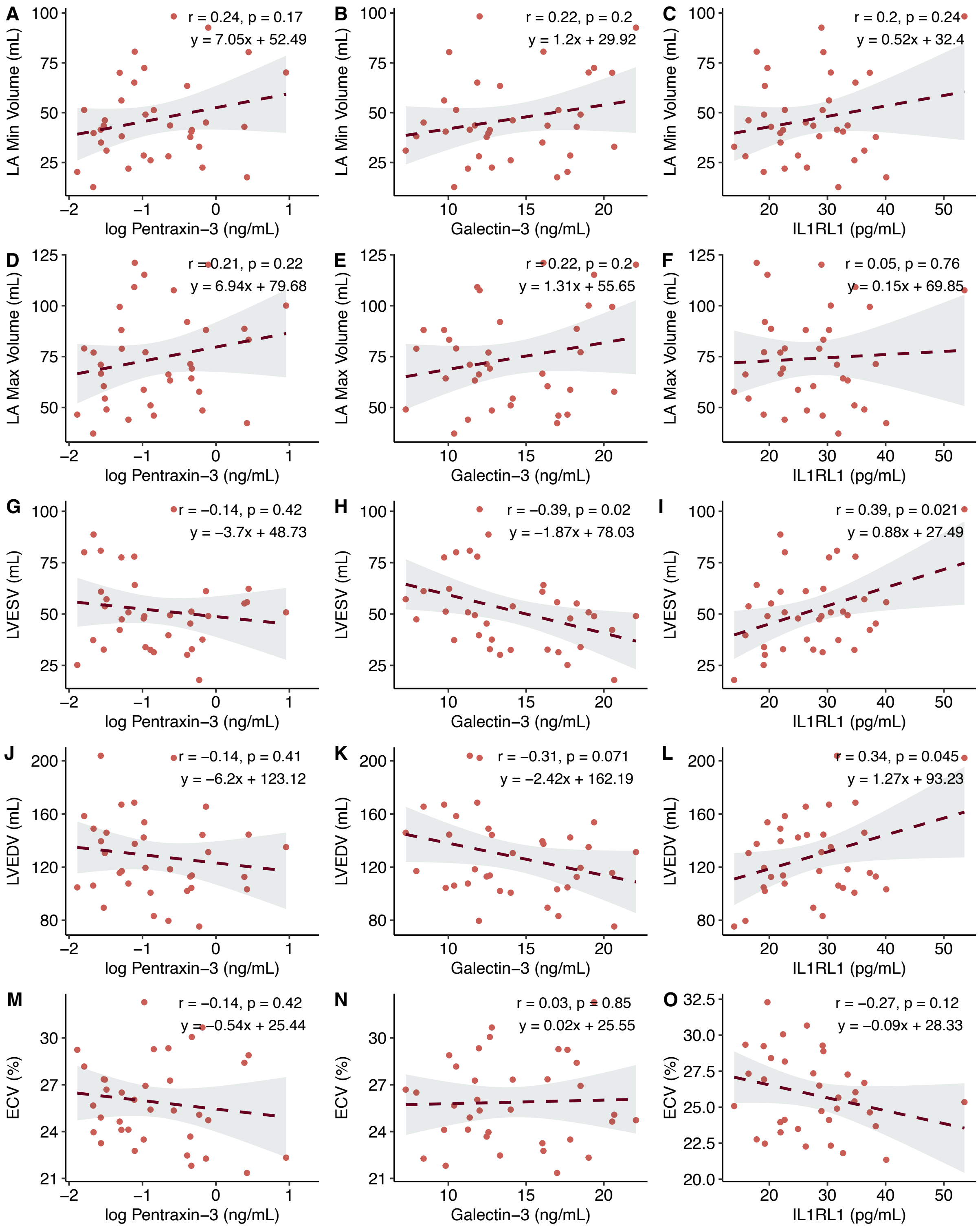

2.2 Pearson's Correlation for Serum Biomarkers and CMR Imaging across all study subjects – continued

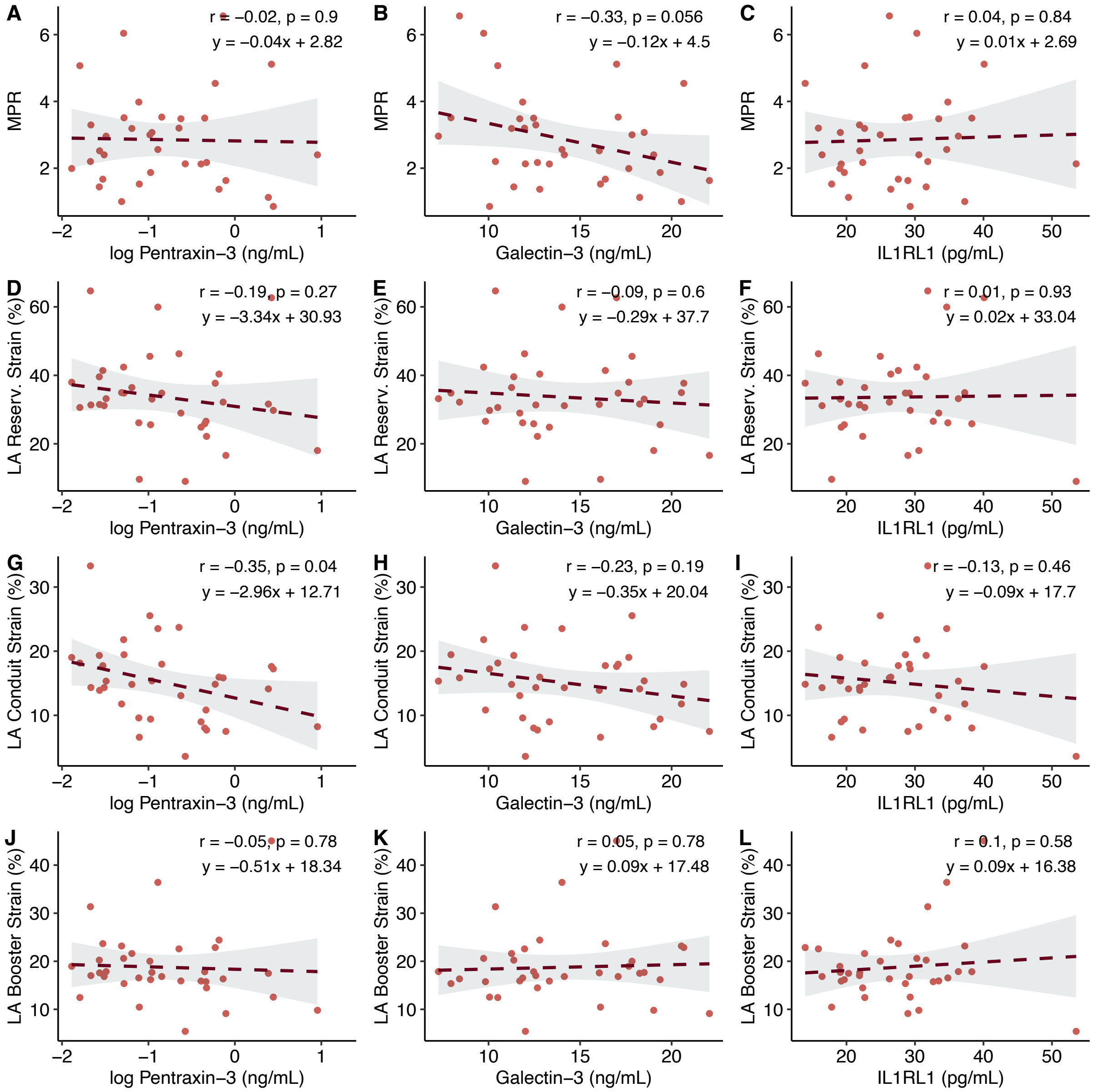

2.3 Pearson's Correlation for Serum Biomarkers and CMR Imaging across HFpEF with T2DM cohort only

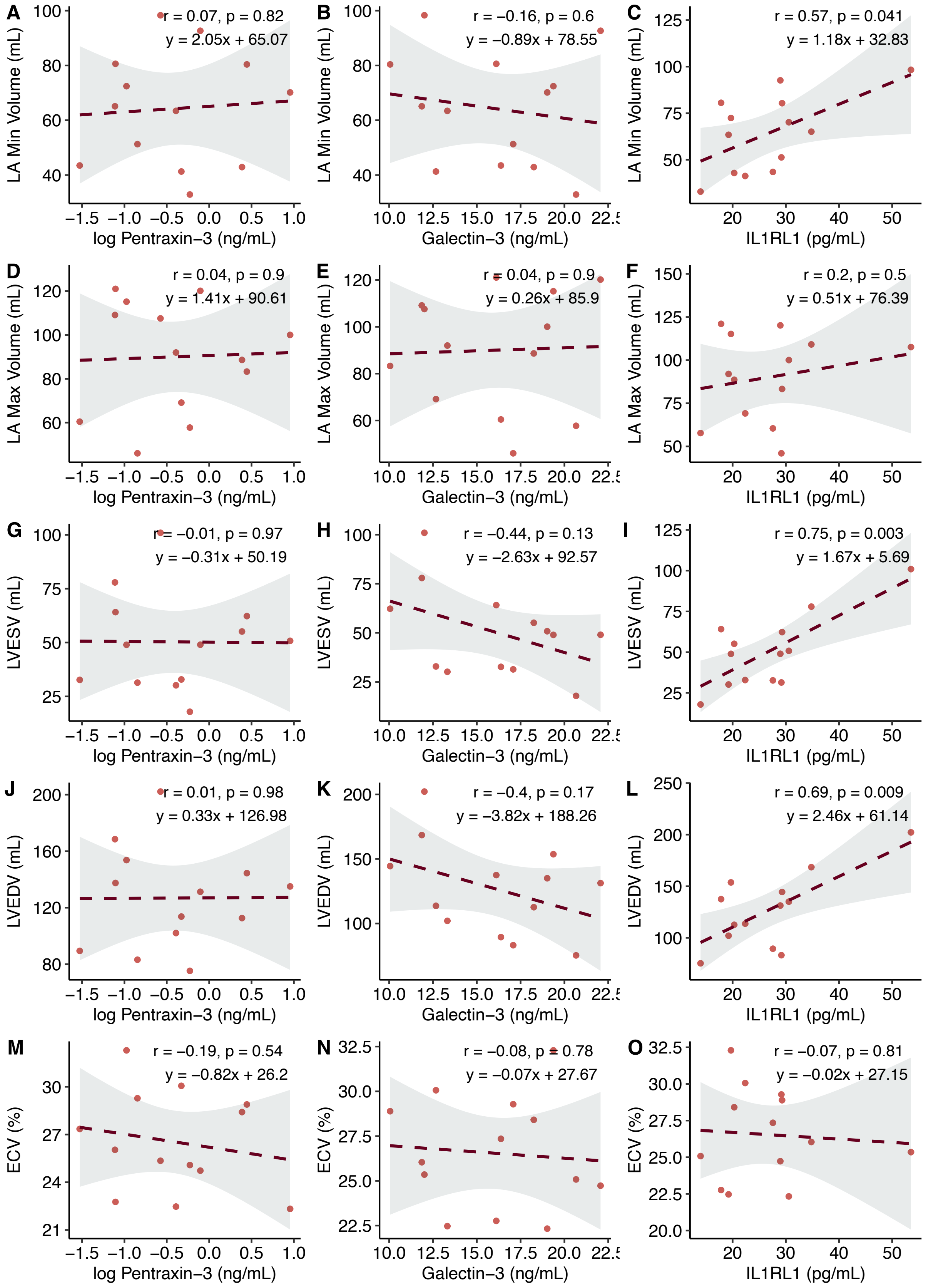

2.4 Pearson's Correlation for Serum Biomarkers and CMR Imaging across HFpEF with T2DM cohort only – continued

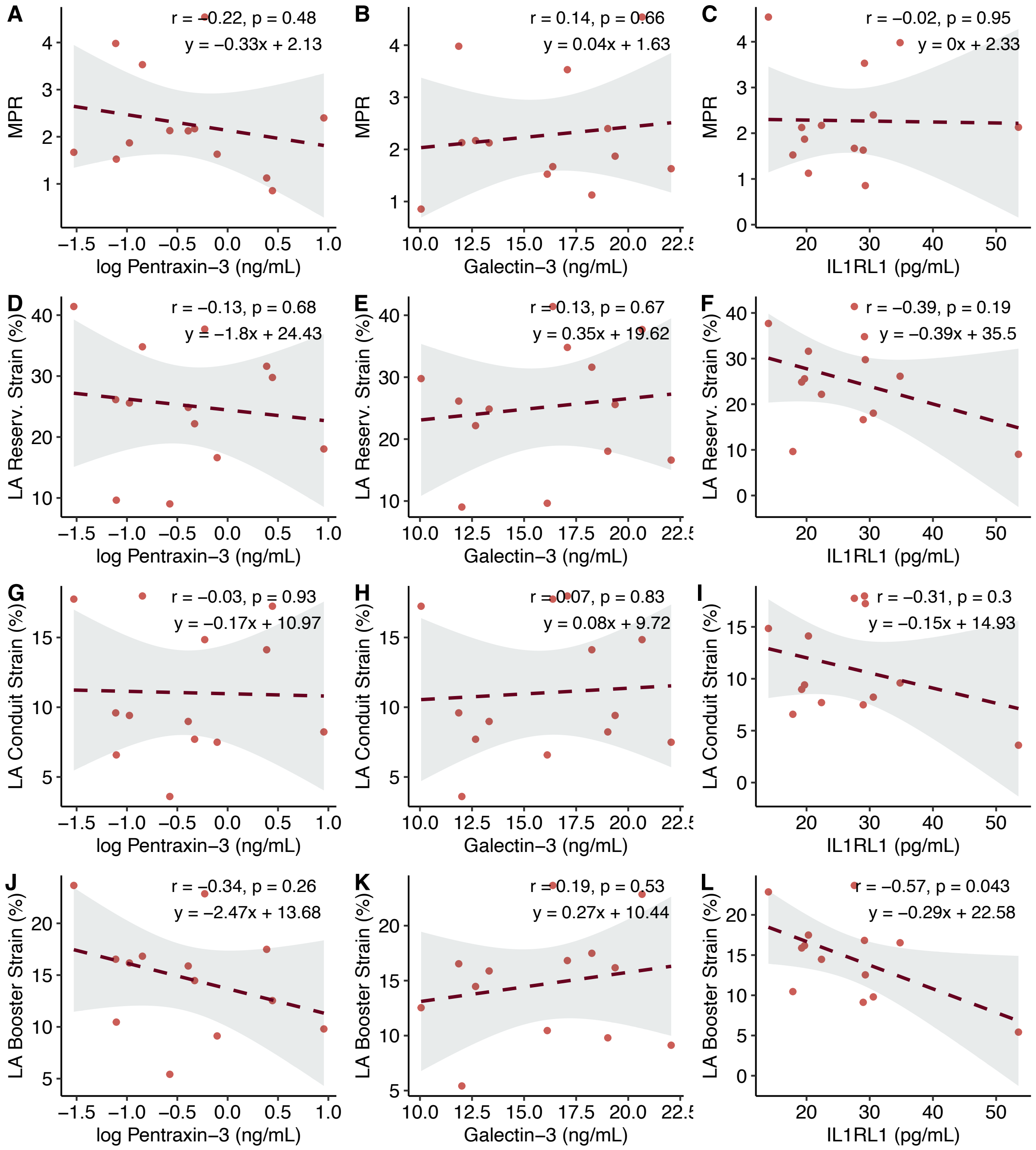

S3. Receiver Operating Curve Analysis

3.1 Visualization

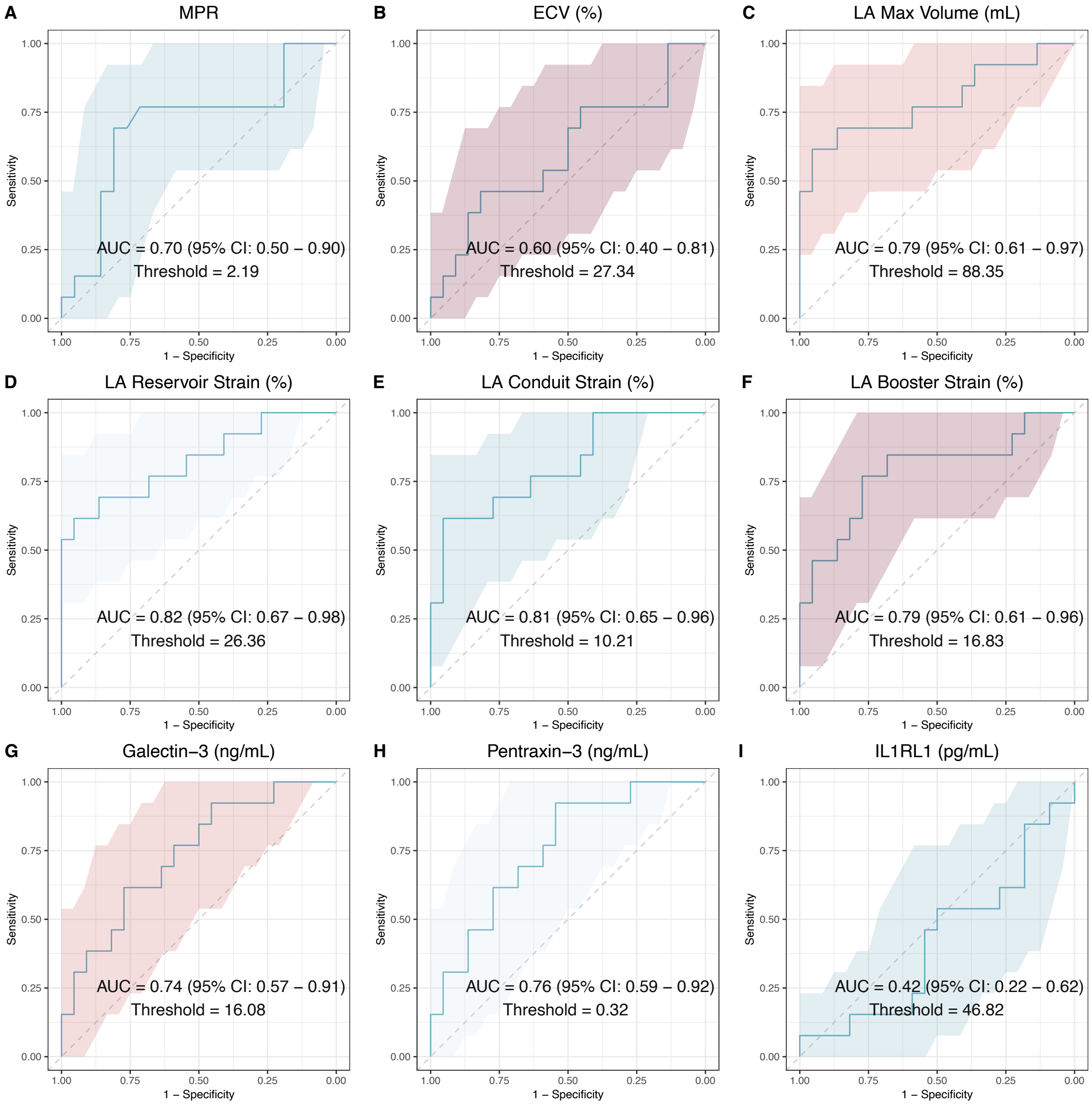

3.2 Summary of Receiver Operating Analysis Statistics

Variable	AUC (95% CI)	Threshold	Sens	Spec	PPV	NPV	Accuracy
**Perfusion & Fibrosis**							
MPR	0.70 (0.50–0.90)	2.19	0.69	0.81	0.69	0.81	0.76
ECV (%)	0.61 (0.40–0.81)	27.34	0.46	0.82	0.60	0.72	0.69
**Function**							
LA Max Vol (mL)	0.79 (0.61–0.97)	88.35	0.62	0.95	0.89	0.81	0.83
LA Reservoir Strain (%)	0.83 (0.67–0.98)	26.36	0.62	0.95	0.89	0.81	0.83
LA Conduit Strain (%)	0.81 (0.65–0.96)	10.21	0.62	0.95	0.89	0.81	0.83
LA Booster Strain (%)	0.79 (0.62–0.96)	16.83	0.77	0.77	0.67	0.85	0.77
**Serum Biomarkers**							
Galectin-3 (ng/mL)	0.74 (0.57–0.91)	16.08	0.62	0.77	0.62	0.77	0.71
Pentraxin-3 (ng/mL)	0.76 (0.59–0.92)	0.32	0.92	0.55	0.55	0.92	0.69
IL1RL1 (pg/mL)	0.42 (0.22–0.62)	46.82	0.08	1.00	1.00	0.65	0.66

AUC = Area under the curve. CI = Confidence interval. ECV = Extracellular volume fraction. Galectin-3 = Galectin-3. IL1RL1 = Interleukin-1 receptor-like 1. LA Booster Strain = Left atrial booster strain. LA Conduit Strain = Left atrial conduit strain. LA Max Vol = Left atrial maximum volume. LA Reservoir Strain = Left atrial reservoir strain. mL = Millilitres. MPR = Myocardial perfusion reserve. ng = Nanograms. NPV = Negative predictive value. pg = Picograms. Pentraxin-3 = Pentraxin-3. PPV = Positive predictive value. Sens = Sensitivity. Spec = Specificity.

Sensitivity Analyses for subgroup pf patients without medical history for atrial arrhythmia

S4. Sensitivity analysis for subgroup without atrial arrhythmia

4.1 Patient Characteristics for subgroup of patients without medical history for atrial arrhythmia

Variable	n	**Obese without CVD n = 6[1]**	**T2DM n = 16[1]**	**HFpEF with T2DM n = 10[1]**	**p-value[2]**
**Demographics**					
Age (years)	32	62 ± 2	61 ± 11	68 ± 4	0.178
Female	32	2 (33%)	1 (6.3%)	3 (30%)	0.191
Body Mass Index (kg/m^2)	32	27.7 ± 1.3	30.6 ± 3.1	31.9 ± 4.6	0.071
**Comorbidities**					
Arterial Hypertension	32	0 (0%)	12 (75%)	10 (100%)	**<0.001**
Hypercholesterolemia	32	0 (0%)	3 (19%)	0 (0%)	0.191
Diabetic Burden (years)	26	NA ± NA	7.9 ± 6.4	11.9 ± 11.5	0.268
Atrial Fibrillation	32	0 (0%)	0 (0%)	0 (0%)	-
Atrial Ectopic Tachycardia	32	0 (0%)	0 (0%)	0 (0%)	-
**Medication**					
ACE inhibitors	35	0 (0%)	5 (31%)	3 (30%)	0.291
Angiotensin II Receptor Blockers	35	0 (0%)	7 (44%)	6 (60%)	0.057
Beta Blockers	35	0 (0%)	3 (19%)	4 (40%)	0.158
Calcium Channel Blockers	35	0 (0%)	7 (44%)	4 (40%)	0.142
SGLT2 Inhibitors	35	0 (0%)	5 (31%)	3 (30%)	0.291
GLP-1 Receptor Agonists	35	0 (0%)	4 (25%)	1 (10%)	0.299
Oral antidiabetics	35	0 (0%)	16 (100%)	10 (100%)	**<0.001**
Insulins	35	0 (0%)	3 (19%)	0 (0.0%)	0.191
Statins	35	0 (0%)	8 (50%)	3 (30%)	0.084
Other lipid-lowering agents	35	0 (0%)	2 (13%)	2 (20%)	0.504
Diuretics	35	0 (0%)	5 (31%)	3 (30%)	0.291

Anticoagulants	35	0 (0%)	0 (0%)	1 (10%)	0.321
Antiplatelets	35	0 (0%)	4 (25%)	2 (20%)	0.406
**Labs**					
Hemoglobin (g/dL)	32	14.53 ± 1.17	14.46 ± 1.13	13.78 ± 1.21	0.296
Total Protein (g/dL)	32	7.15 ± 0.40	7.31 ± 0.28	7.21 ± 0.41	0.545
Creatinine (mg/dL)	32	0.87 ± 0.14	0.99 ± 0.21	1.08 ± 0.26	0.181
Hemoglobin A1c (%)	16	5.23 ± 0.29	6.29 ± 1.53	6.53 ± 0.85	**0.041**
NT-proBNP (pg/mL)	31	6 ± 2	6 ± 3	48 ± 26	**<0.001**
**Cardiac Magnetic Resonance**					
LVEDV (mL)	32	133 ± 27	127 ± 30	122 ± 32	0.773
LVSV (mL)	32	79 ± 9	75 ± 23	76 ± 17	0.915
LVEF (%)	32	61 ± 9	59 ± 9	63 ± 7	0.488

[1] Mean ± SD; n (%)

[2] One-way ANOVA; Pearson’s Chi-squared test

4.2 Findings for subgroup pf patients without medical history for atrial arrhythmia

Variable	n	Obese without CVD n = 6[1]	T2DM n = 16[1]	HFpEF with T2DM n = 10[1]	p-value[2]
**Perfusion & Fibrosis**					
MPR	31	3.63 ± 1.62	3.05 ± 1.41	2.41 ± 1.20	0.239
ECV (%)	32	26.73 ± 1.81	25.03 ± 2.65	26.12 ± 3.32	0.377
**Atrial Morphology & Function**					
Minimum LA Volume (mL)	32	35 ± 17	37 ± 13	65 ± 18	**<0.001**
Maximum LA Volume (mL)	32	63 ± 21	65 ± 16	91 ± 28	**0.011**
LA Reservoir Strain (%)	32	41 ± 12	38 ± 11	26 ± 10	**0.021**
LA Conduit Strain (%)	32	21.2 ± 6.3	16.1 ± 4.8	11.8 ± 4.6	**0.004**
LA Booster Strain (%)	32	19 ± 7	22 ± 8	15 ± 5	0.093
**Serum Biomarkers**					
Galectin-3 (ng/mL)	32	10.6 ± 3.7	13.8 ± 3.3	16.6 ± 3.9	**0.010**
Pentraxin-3 (ng/mL)	32	0.21 ± 0.05	0.52 ± 0.35	0.82 ± 0.74	0.063
Interleukin-1 Receptor-Like 1 (pg/mL)	32	28 ± 6	28 ± 8	25 ± 7	0.619

[1] Mean ± SD; n (%)
[2] One-way ANOVA

4.3 Logistic Regression Analyses for subgroup of patients without medical history for atrial arrhythmia

	Univariable Logistic Regression		Multivariable Logistic Regression*	
**Variable**	**OR (95% CI)**	**p-value**	**OR (95% CI)**	**p-value**
**Hemodynamics & Fibrosis**				
ECV (%)	1.09 (0.82, 1.45)	0.545	-	-
MPR	0.61 (0.28, 1.11)	0.150	-	-
**Function**				
LV EDV (mL)	0.99 (0.96, 1.02)	0.538	-	-
LA Max Vol (mL)	1.06 (1.02, 1.11)	**0.011**	1.21 (1.04, 1.27)	**0.015**
LA Reservoir Strain (%)	0.85 (0.72, 0.95)	**0.022**	0.71 (0.44, 0.91)	**0.047**
LA Conduit Strain (%)	0.77 (0.60, 0.93)	**0.017**	0.58 (0.32, 0.85)	**0.024**
LA Booster Strain (%)	0.80 (0.61, 0.96)	**0.045**	0.72 (0.49, 0.93)	**0.034**
**Serum Biomarkers**				
Galectin-3 (ng/mL)	1.29 (1.05, 1.67)	**0.025**	1.26 (0.98, 1.71)	0.097
Pentraxin-3 (ng/mL)[†]	3.21 (1.06, 12.2)	0.055	-	-
IL1RL1 (pg/mL)	0.95 (0.84, 1.05)	0.317	-	-

*Included the respective variable significant in univariable logistic regression and additionally accounted for age, sex and BMI.
[†] Values for log-transformed Pentraxin-3.

4.4 Comparative analysis of Serum Biomarkers and CMR Imaging across Subgroup of patients without medical history for atrial arrhythmia

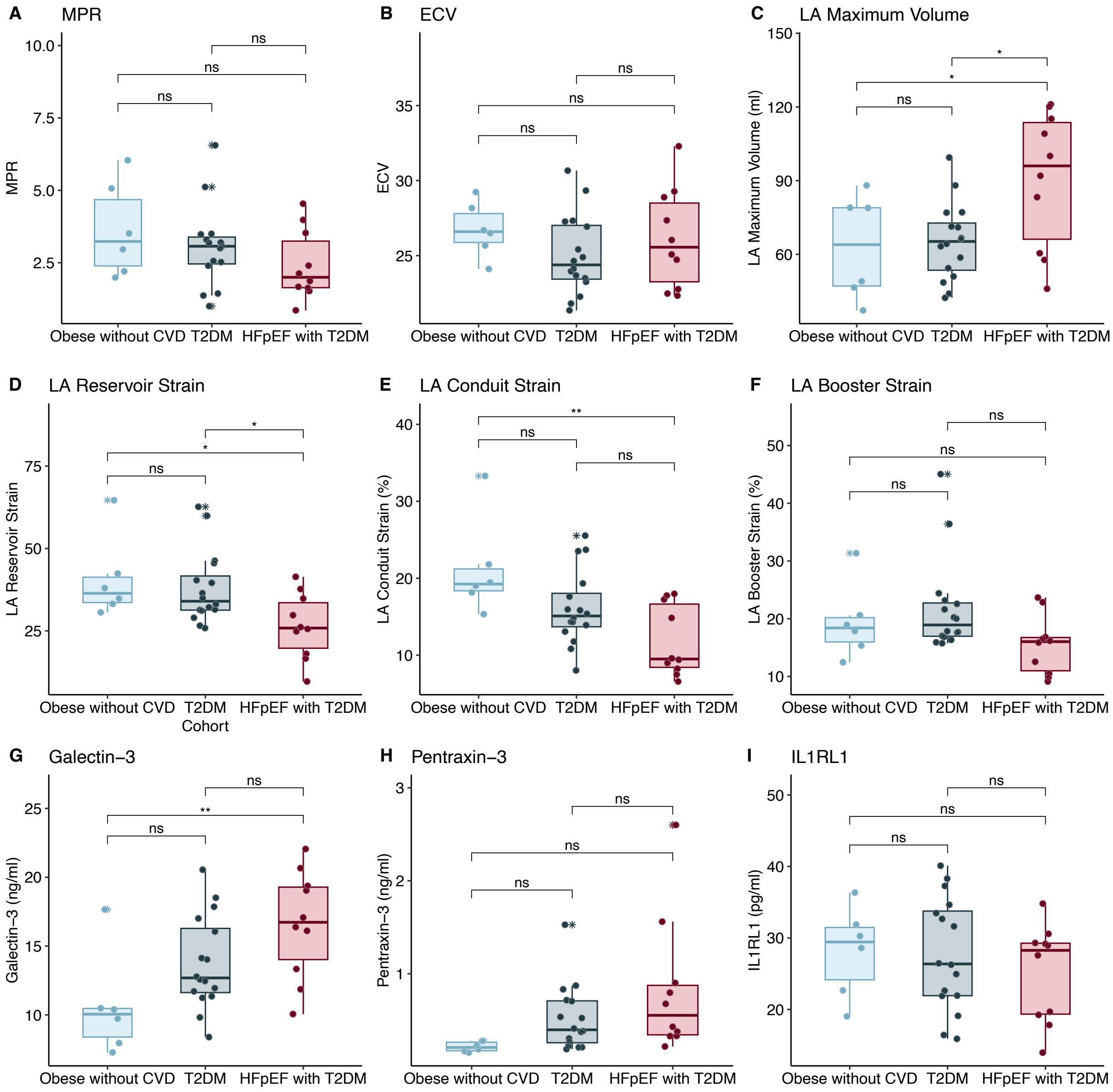

4.5 Pearson's Correlation for Serum Biomarkers and CMR Imaging across Subgroup of patients without medical history for atrial arrhythmia

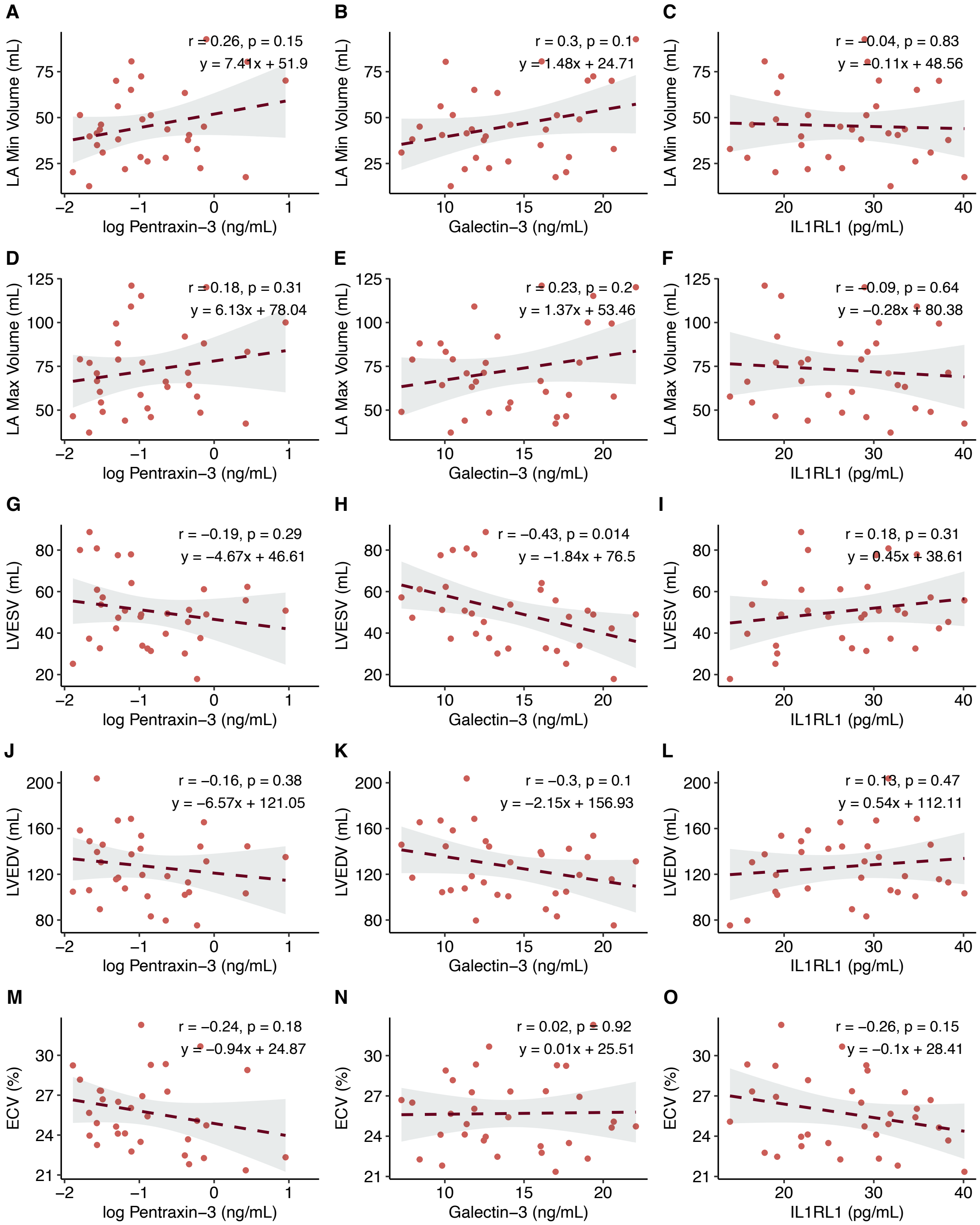

4.6 Pearson's Correlation for Serum Biomarkers and CMR Imaging Subgroup of patients without medical history for atrial arrhythmia – continued

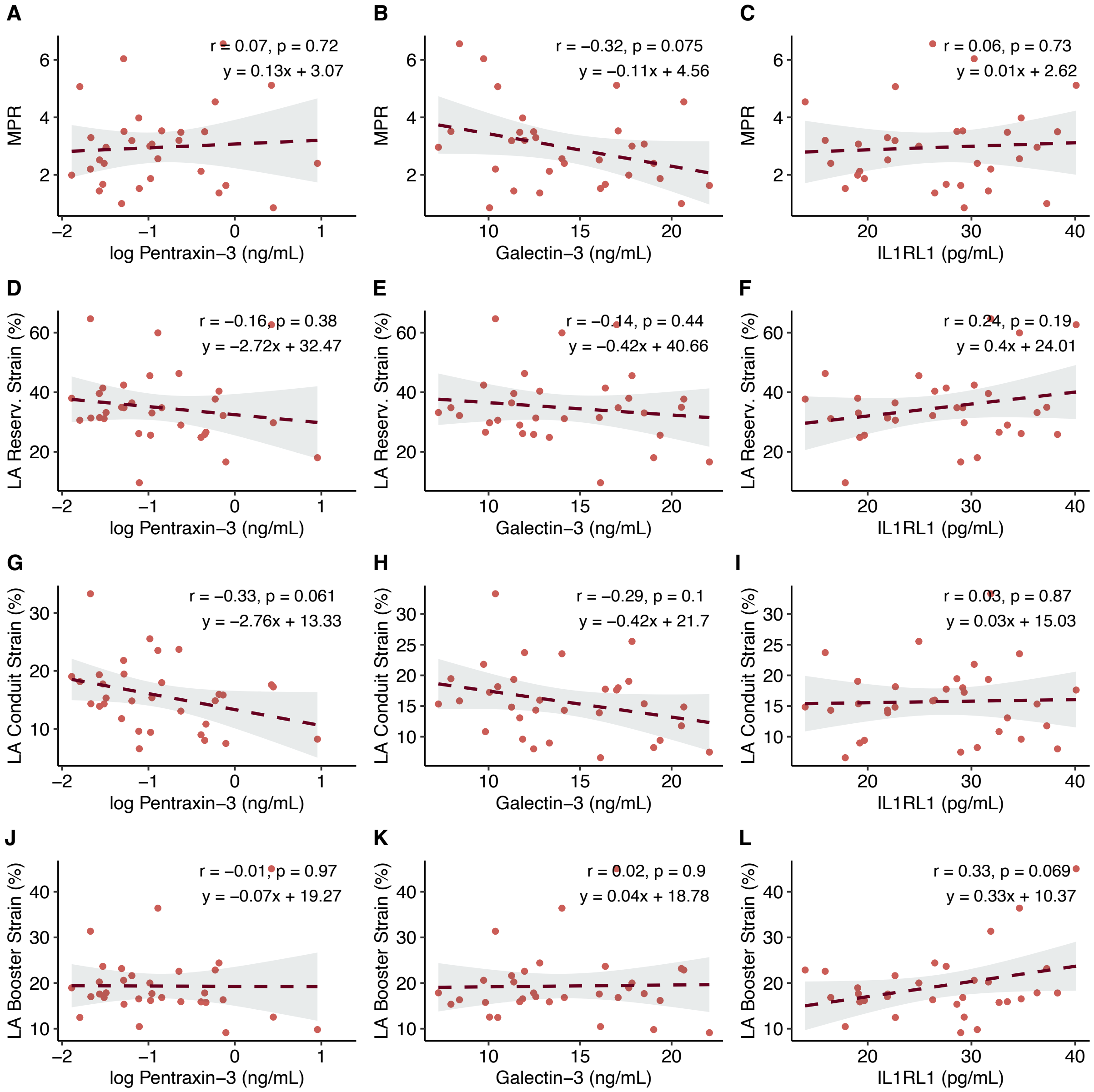

4.7 Pearson's Correlation for Serum Biomarkers and CMR Imaging across HFpEF with T2DM cohort without medical history for atrial arrhythmia only

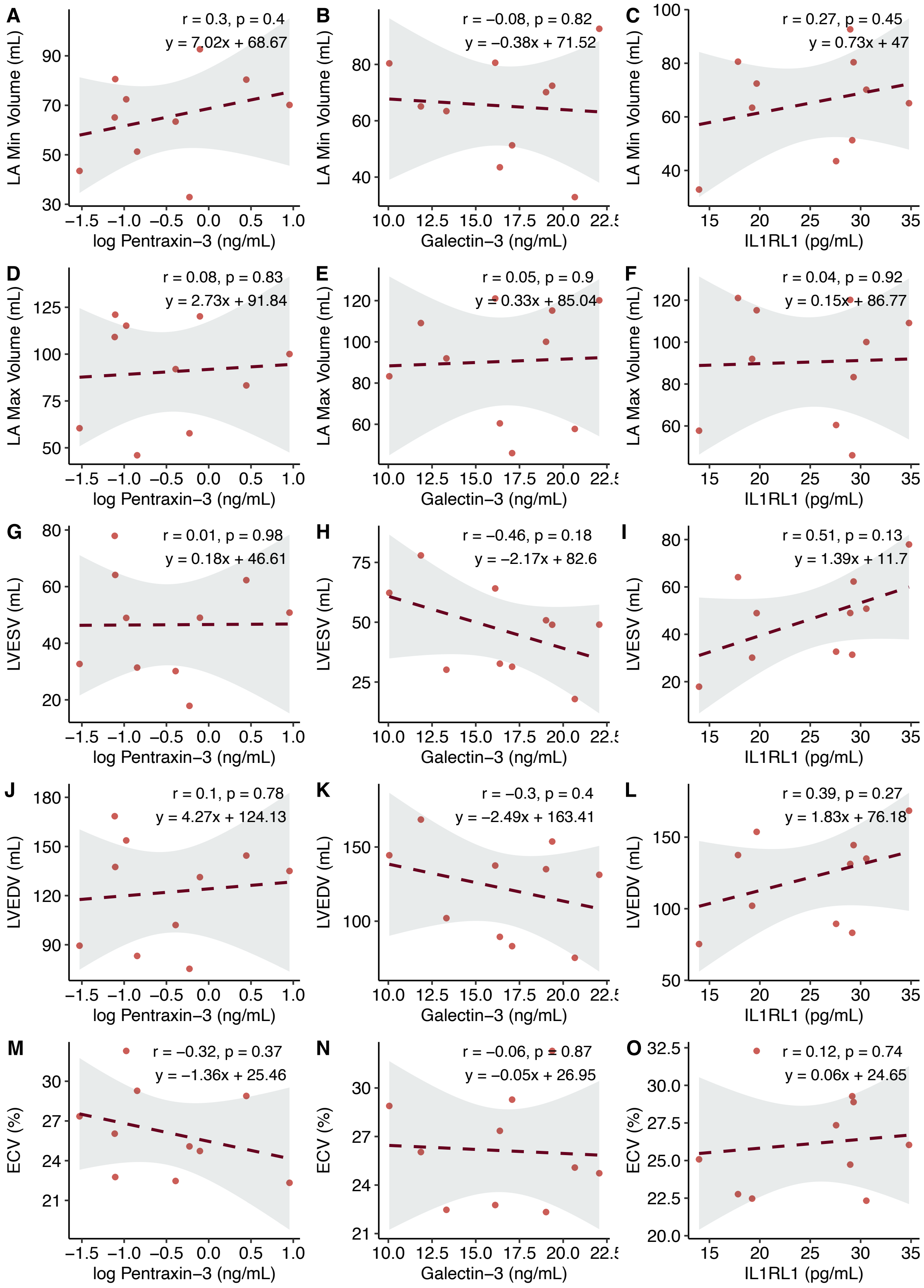

4.8 Pearson's Correlation for Serum Biomarkers and CMR Imaging across HFpEF with T2DM cohort without medical history for atrial arrhythmia only – continued

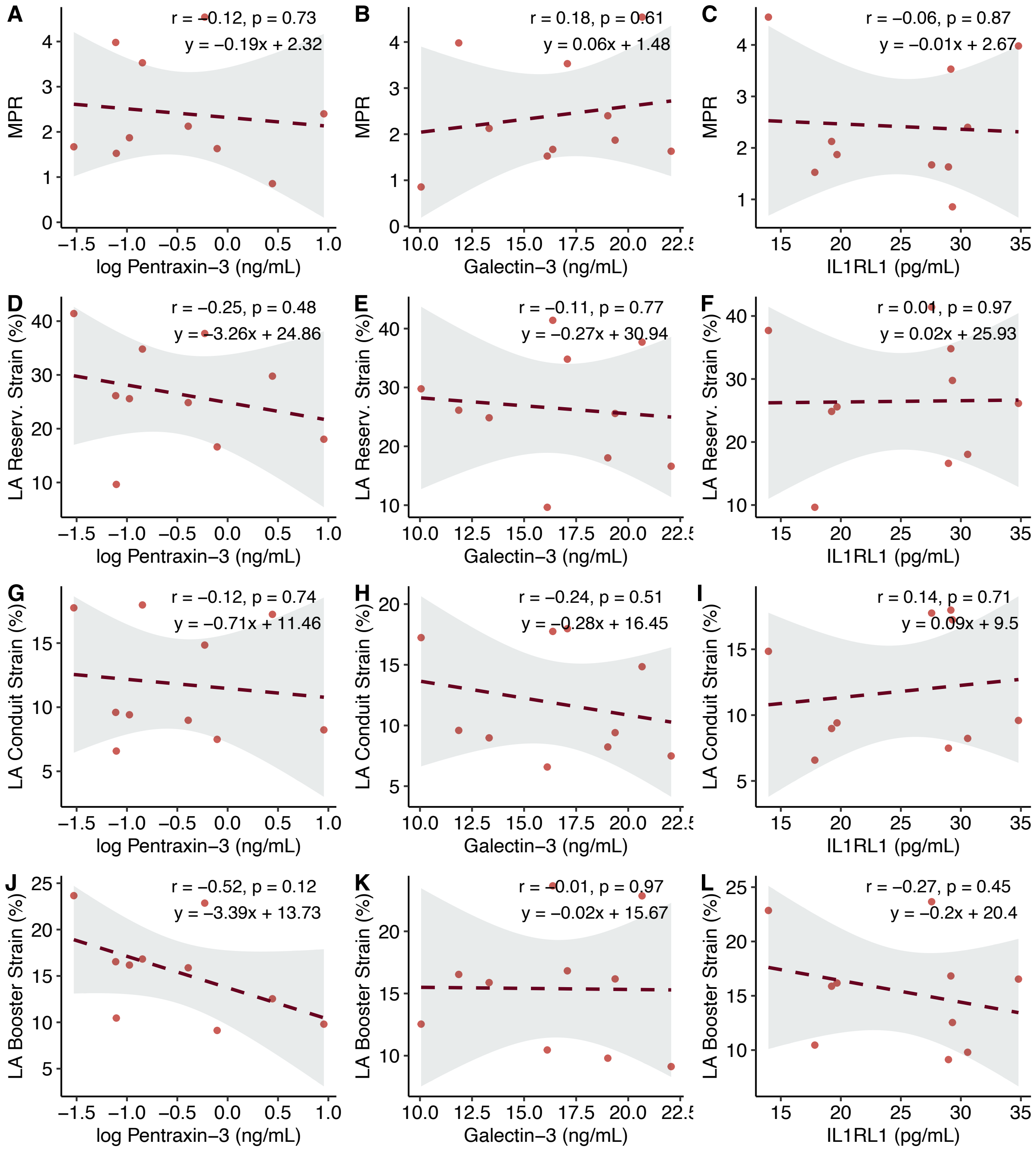

4.9 Visualization of Receiver Operating Analysis across HFpEF with T2DM cohort without medical history for atrial arrhythmia only

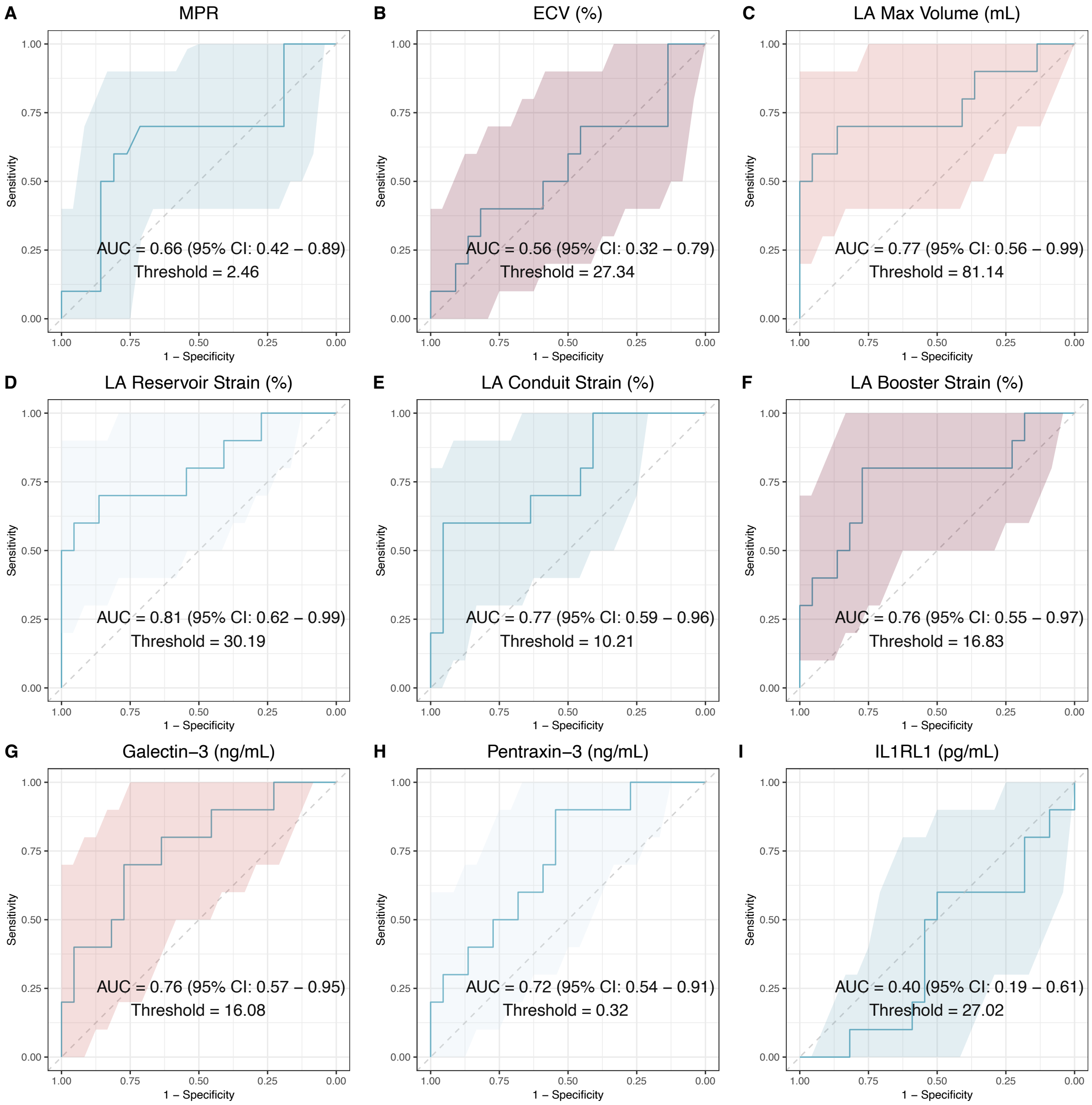

4.10 Summary of Receiver Operating Analysis Statistics across HFpEF with T2DM cohort without medical history for atrial arrhythmia only

Variable	AUC (95% CI)	Threshold	Sens	Spec	PPV	NPV	Accuracy
**Perfusion & Fibrosis**							
MPR	0.66 (0.42–0.89)	2.46	0.70	0.71	0.54	0.83	0.71
ECV (%)	0.56 (0.32–0.79)	27.34	0.40	0.82	0.50	0.75	0.69
**Function**							
LA Max Vol (mL)	0.77 (0.56–0.99)	81.14	0.70	0.86	0.70	0.86	0.81
LA Reservoir Strain (%)	0.81 (0.62–0.99)	30.19	0.70	0.86	0.70	0.86	0.81
LA Conduit Strain (%)	0.77 (0.59–0.96)	10.21	0.60	0.95	0.86	0.84	0.84
LA Booster Strain (%)	0.76 (0.55–0.97)	16.83	0.80	0.77	0.62	0.89	0.78
**Serum Biomarkers**							
Galectin-3 (ng/mL)	0.76 (0.57–0.95)	16.08	0.70	0.77	0.58	0.85	0.75
Pentraxin-3 (ng/mL)	0.72 (0.54–0.91)	0.32	0.90	0.55	0.47	0.92	0.66
IL1RL1 (pg/mL)	0.40 (0.19–0.61)	27.02	0.60	0.50	0.35	0.73	0.53

AUC = Area under the curve. CI = Confidence interval. ECV = Extracellular volume fraction. Galectin-3 = Galectin-3. IL1RL1 = Interleukin-1 receptor-like 1. LA Booster Strain = Left atrial booster strain. LA Conduit Strain = Left atrial conduit strain. LA Max Vol = Left atrial maximum volume. LA Reservoir Strain = Left atrial reservoir strain. mL = Millilitres. MPR = Myocardial perfusion reserve. ng = Nanograms. NPV = Negative predictive value. pg = Picograms. Pentraxin-3 = Pentraxin-3. PPV = Positive predictive value. Sens = Sensitivity. Spec = Specificity.
